# Bipolar and Unipolar Silylene-Diphenylene σ-π Conjugated Polymer Route for Highly Efficient Electrophosphorescence

**DOI:** 10.1038/srep38404

**Published:** 2016-12-02

**Authors:** Yao-Tang Chang, Sunil Sharma, Miao-Ken Hung, Yu-Hsuan Lee, Show-An Chen

**Affiliations:** 1 Department of Chemical Engineering and Frontier Research Center on Fundamental and Applied Sciences of Matters, National Tsing-Hua University, Hsinchu 30013, Taiwan (ROC)

## Abstract

σ-π conjugated polymer strategy is proposed for designing electroluminescent host polymers with silylene-diphenylene as the backbone repeat unit giving a high triplet energy (E_T_ = 2.67 eV). By incorporation of high E_T_ (3.0 eV) electron (oxadiazole, OXD) and hole (triphenyl amine, TPA) transport moieties, or TPA alone (in this case, the main chain acts as electron transport channel) as side arms on the silylene, the high E_T_ bipolar and unipolar polymers are formed, allowing a use of iridium green phosphor (Ir(ppy)_2_(acac), Ir-G) (E_T_ = 2.40 eV) as the dopant. The matching of energy levels of the dopant with the hosts, leading to charge trapping into it; and singlets and triplets of the exciplex and excimer can be harvested via energy transfer to the dopant. Using these host-guest systems as the emitting layer, chlorinated indium-tin-oxide (Cl-ITO) as the anode, and benzimidazole derivative (TPBI) as the electron transport layer, this two-layer device gives the high luminance efficiency 80.1 cd/A and external quantum efficiency 21.2%, which is the best among the report values for polymer light emitting diode (PLED) in the literatures. This example manifests that σ-π conjugated polymer strategy is a promising route for designing polymer host for efficient electrophosphorescence.

Conventional polymers for use as emitter in light emitting diode (LED) have been studied extensively due to its capability for large area device, low cost fabrication process, and simplicity in device structure. One of the main problems in this type of polymer LED (PLED) is that the triplet energy (E_T_) of the polymer backbone is not high enough when used as a host for phosphor dopant such as iridium complexes. In such host/guest system, both singlet and triplet excitons can be harvested and consequently the internal quantum efficiency is able to be promoted to 100% since the heavy-metal complexes can induce strong spin-orbital coupling and thus promote efficient intersystem crossing from its singlet excited state to the lowest-energy triplet excited state of the guest, and finally followed by relaxation to the ground state via phosphorescence^1^,^2^,^3^. The lowering in E_T_ of conventional conjugated polymer is resulted from extensive π-electron delocalization along the main chain. For examples, 2.18 eV for polyfluorenes and 2.27 eV for poly(p-phenylene)s^3^ are too low relative to the green (2.4 eV) and blue (2.65 eV) iridium phosphor dopants, respectively, leading to a low emitting efficiency by back transfer of E_T_ from the phosphors to the polymer hosts in their corresponding devices^4^.

So far, some efforts have been attempted to overcome the problems of low E_T_ of polymer host in PLED by integrating conjugated bipolar transport moieties spaced with oxygen to break the conjugation along the backbone and grafting phosphor as side chain (similar to that in simultaneous red-green-blue emission polyfluorenes)^5^ for sky blue emission with the high efficiency 9% in EQE from a three-layer device^6^. However, introducing oxygen as the spacer leads to lower charge mobility as reflected from its lower current density (below 0.1 A/cm^2^) and brightness (maximum at 5500 cd/m^2^) relative to those of normal OLED and PLED at about 0.5~1.5 A/cm^2^ and 20000~50000 cd/m^2^, respectively^7^,^8^. For non-conjugated polymer poly(vinylcarbazole) (PVK), which is generally used as host, when OXD-grafted non-conjugated polymer is used as a cohost, its maximum luminance efficiency and external quantum efficiency (EQE) of green phosphorescence device are able to reach 44.6 cd/A and 13.6%, respectively, with high turn on voltage (>5.0 V) and moderate maximum brightness 10,000 cd/m^2^ resulting from its lower current density (also below 0.1 A/cm^2^)^9^. Very recently, alternative-current-driven devices with PVK as host were developed, exhibiting the high luminance efficiency 110 cd/A from a four-layer device; but, due to the higher operating voltage (±75 V), its corresponding maximum power efficiency was only 29.3 lm/W^10^.

Unlike the conventional π-π conjugated polymers, The σ-π conjugated polymer is a promising structure to give high triplet energy. In the past, the reports for σ-π conjugation phenomena usually focus on their energy change in photoluminescence and absorption spectra^11^,^12^, and the change in electrochemical oxidation potential^13^. Moreover, the studies of σ-π conjugated polymers in optoelectronics are very rare. The reported σ-π conjugated materials consist of oligosilylene (σ-part) and π-conjugated (π-part) moieties, and exhibit both properties of them^14^. The oligosilylene part possesses great hole mobility (~10^−4^ cm V^−2^ s^−1^)^15^,^16^ and rather high band gap (>3.5 eV)^17^, while the π-conjugated part can facilitate electron transport ability. However, the Si-Si bonding of silylene is unstable under UV irradiation^17^, and longer π-conjugated length also gives lower E_T_. Thus, for an ideal σ-π conjugated material applied to optoelectronics, it should be avoided to introduce Si-Si bonding and π-conjugated length should be limited to two phenyl rings or less (biphenyl gives reasonably high E_T_ 2.85 eV)^18^ while p-terphenyl gives an E_T_ of 2.55 eV, which is too low for blue phosphor emitter guest.

Here, we design the σ-π conjugated polymers, by incorporation of electron (oxadiazole, OXD) and hole (triphenyl amine, TPA) transport moieties for bipolar polymer and TPA only for unipolar polymer as side arms on silylene in the silylene diphenylene backbone. Upon doping with green emission Ir-complex, (Ir(ppy)_2_(acac), Ir-G), along with the cholorinated indium-tin-oxide (Cl-ITO)^19^,^20^ glass plate as the anode, 2,2′,2″-(1,3,5-benzinetriyl)-tris(1-phenyl-1-H- benzimidazole) (TPBI) as the electron transport layer, the two-layer-only device gives the extremely high performance with maximum luminance efficiency 80.1 cd/A (EQE 21.2%), corresponding power efficiency 62.9 lm/W) and maximum brightness 25452 cd/m^2^, which is the best among the report values for PLED in the literatures. For both unipolar and bipolar polymers, two separated channels are permitted for electron and hole transport toward the dopant by charge trapping. For the former, electrons and holes are transport via OXD and TPA moieties, respectively; while for the later, electrons are via main chain and holes via TPA. Furthermore, the formation of TPA-OXD exciplex in the bipolar polymer and that of TPA excimer in the unipolar polymer can also provide an additional route for transferring exciton energy to Ir-G. For comparison of the present results with those reported in the literatures described above, all these results are summarized in Table 1.

## Results and Discussion

### σ-π conjugated polymers: synthesis and optical/semiconductor characterization

**Figure 1:**
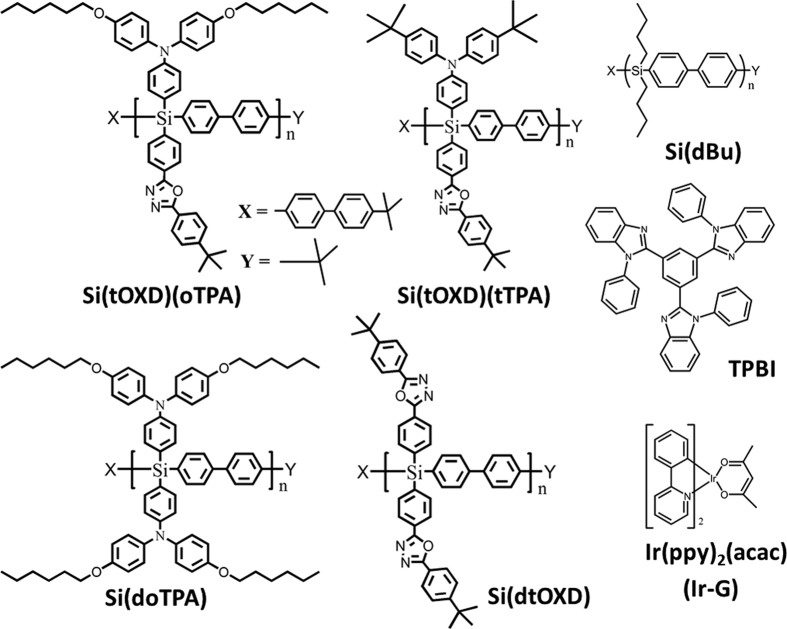
Molecular structures of the proposed σ-π conjugated polymers.

For demonstrating the present σ-π conjugated molecular design strategy for use as host in electroluminescence, we synthesize poly(dibutylsilylene-diphenylene) [Si(dBu)] as the main chain analogue (see Fig. 1), in which its backbone is the same as the proposed polymers but the transport moieties attached to Si are replaced by butyl groups. Compared to the absorption peak of biphenyl molecule at 242 nm, that of Si(dBu) shows red-shift to 271 nm by 29 nm (Supplementary Fig. 1a), which is resulted from a hyper-conjugative manner between benzene π orbitals and silicon σ orbitals, which forms a low-lying σ_π_ (σ_π_*) orbitals to stabilize the structure^21^,^22^. Furthermore, in single carrier measurement (Supplementary Fig. 1b), its hole mobility of 2.76 × 10^−7^ cm^2^ V^−1^ s^−1^ and electron mobility of 8.47 × 10^−5^ cm^2^ V^−1^ s^−1^ are determined by applying the space-charge limited current (SCLC) method^23^,^24^. The charge mobilities at the level of 10^−5^~10^−7^ cm^2^ V^−1^ s^−1^ indicate that the σ-π conjugated silylene-diphenylene backbone possesses semiconducting characteristic and can provide as one of the carriers transport pathways.

Herein, we synthesized four transport moieties modified silylene-diphenylene polymers containing two unipolar materials, Si(doTPA) and Si(dtOXD), and two bipolar materials Si(tOXD)(oTPA) and Si(tOXD)(tTPA), where TPA is triphenyl amine, OXD is oxadiazole, and “d” denotes the di-substitution, and “t” and “o” denote tert-butyl and alkoxy, respectively. The chemical structures of these host polymers, guest Ir(ppy)_2_(acac) and electron transport layer material TPBI are given in Fig. 1. The synthesis and characterization details are shown in the Supplementary Methods. Their energy levels were measured using cyclic voltammograms (CV) (Supplementary Fig. 2 and Supplementary Table 2), absorption spectroscopy (Supplementary Fig. 3) and ultraviolet photoelectron spectroscopy (UPS) (Supplementary Fig. 4). The molecular weight (Mw) and polydispersity index (PDI) relative to polystyrene standards are 31000 Dalton and 1.99 for Si(doTPA), 12000 Dalton and 1.40 for Si(dtOXD), 48000 Dalton and 2.57 for Si(tOXD)(oTPA), and 77000 Dalton and 2.99 for Si(tOXD)(tTPA).

From ultraviolet-visible (UV-Vis) and photoluminescence (PL) spectroscopy measurements at room temperature (Supplementary Fig. 3 and Supplementary Table 2), the absorption peaks of the four functionalized polymers in dilute solutions (1 × 10^−5^ M in chloroform) or as thin solid films are all at about 290 nm, and all the emission peaks of thin solid films exhibit a similar trend of red-shift by 10–20 nm relative to those from the dilute solutions. The obvious excimer emissions are observed in thin solid films of the unipolar polymers Si(doTPA) and Si(dtOXD) (Supplementary Figs 3a,b and 5). For the bipolar polymers, their emission peaks are significantly red-shifted compared to those of the unipolar polymers, resulting from the formation of exciplex between the electron-rich TPA and electron deficient OXD moieties, which gives lower energy emission than its corresponding excimer emissions (Supplementary Fig. 3c,d). While the four host materials are doped with 8 wt% Ir-G, only green emissions with a peak at 520 nm (Supplementary Fig. 6) are observed, indicating an occurrence of highly efficient energy transfer from the host to the guest.

### Triplet state measurements

**Figure 2:**
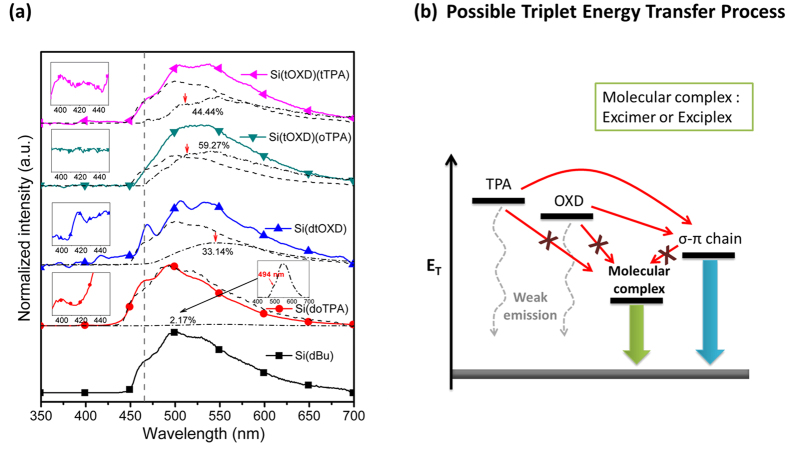
Triplet state measurements and the possible triplet energy transfer process. (**a**) Phosphorescence spectra delayed by 1 ms in diluted toluene solutions of Si(dBu), Si(doTPA), Si(dtOXD), Si(tOXD)(oTPA) and Si(tOXD)(tTPA). The insets are the enlargements for each polymer in the range 390–450 nm. Note: main chain for Si(doTPA), the fraction of triplet excimer emission is trace (2.17%) as indicated by its near overlay with the baseline. Here, the fitting curves are the mainchain analogue (‒ ‒ ‒) and the excimer/exciplex emission (‒ · ‒). The numbers in percentage are the fraction of triplet emission contributed from the molecular complexes: excimer and exciplex obtained by deconvolution of the total triplet emission assuming the contribution from main chain and molecular complexes of the moieties on side arms. (**b**) Schematic diagram of the triplet energy transfer process of the bipolar polymer under photoexcitation at low temperature.

From delayed phosphorescence measurements, all the triplet emission spectra and their characteristic values of the silylene-diphenylene polymer in the dilute solution (5 × 10^−5^ M) at 77 K are shown in Fig. 2a and Table 2, respectively. The detailed measurement procedures are shown in Supplementary Notes. Si(dBu) exhibits phosphorescence peaks at 465(shoulder (sh)), 493 and 533(sh) nm possessing an E_T_ 2.67 eV (calculated from the peak 465 nm). Compared to that of molecular biphenyl 2.85 eV, the lower E_T_ is resulted from the σ-π hyper-conjugation^21^,^22^. For the unipolar polymers, the phosphorescence spectrum of Si(doTPA) with the peaks at 465(sh), 493 and 533(sh) nm and that of Si(dtOXD) with the peaks at 468, 503 and 533(sh) nm are quite similar to that of Si(dBu). Also, for the unipolar polymers, by enlarging the spectra in the range 390–450 nm, a peak of Si(doTPA) at 405 nm and two weak peaks of Si(dtOXD) at 416 and 446 nm are observed, which are nearly identical to their corresponding isolated TPA at 405 nm and OXD at 414 and 446 nm^25^,^26^, indicating that the occurrence of triplet energy transfer from the side arm TPA or OXD moieties to the backbone silylene-diphenylene is efficient but still incomplete. As the side arm moieties can form excimer or exciplex, their triplet emissions should also appear in the broad peaks from 450 to 700 nm in addition to that from the main chains. We further performed the spectral deconvolutions by fitting each spectrum with the spectral distributions of Si(dBu) and molecular complex in Fig. 2a, and found that the phosphorescence of the unipolar polymers Si(doTPA) and Si(dtOXD) possess 2.17% contribution from TPA excimer and 33.14% contribution from OXD excimer emissions, respectively. Up to this point, we found that the present σ-π polymers containing multiple triplet states, contributing from: isolated transport moieties, molecular complexes, and main chain. Thus the E_T_s of Si(doTPA) are assigned as 3.07 eV (referred to the peak 405 nm) for side arm isolated TPA, 2.67 eV (referred to the shoulder 465 nm) for silylene-diphenylene backbone and 2.52 eV (referred to the shoulder 494 nm) for TPA excimer (Supplementary Fig. 7a). Also, E_T_s of Si(dtOXD) are 2.98 eV (referred to the peak 416 nm) for side arm isolated OXD, 2.66 eV (referred to the peak 468 nm) for silylene- diphenylene backbone and 2.28 eV (referred to the peak 546 nm) for OXD excimer (the presence of OXD excimer is revealed in Supplementary Fig. 7b). For the bipolar polymers, both Si(tOXD)(oTPA) and Si(tOXD)(tTPA) show the same phosphorescence peaks at 467(sh), 503 and 536 nm. By enlarging their spectra in the range 390–450 nm, only Si(tOXD)(tTPA) shows peaks at 405 and 424 nm contributed by both isolated TPA and OXD moieties, but Si(tOXD)(oTPA) shows no observable peak. This difference is probably due to the more steric hindrance of tTPA than oTPA in forming exciplex with tOXD, leading to less isolated tOXD and TPA in Si(tOXD)(oTPA) than in Si(tOXD)(tTPA); such that after triplet energy transfer from side arms to backbone, Si(tOXD)(tTPA) has more amount of isolated OXD and TPA than Si(tOXD)(oTPA). On the other hand, by subtracting the normalized Si(dBu) spectrum from the normalized Si(tOXD)(oTPA) and Si(tOXD)(tTPA) spectra individually at the first peak of Si(dBu) 465 nm, both the residual spectra are very similar (Supplementary Fig. 8) with the peaks at 513 and 547 nm resulting from TPA-OXD exciplex triplet emissions, which gives an E_T_ of 2.42 eV referred to the peak 513 nm. By such deconvolution, the fractions of triplet exciplex emission are 59.27% and 44.44% for Si(tOXD)(oTPA) and Si(tOXD)(tTPA), respectively. This result also in agreement with no residual triplet emission from tOXD and oTPA in Si(tOXD)(oTPA) discussed above.

As the present σ-π polymers show multiple triplet states, the energy transfer routes among them are shown in Fig. 2b and depicted below. The side arm isolated moieties TPA (3.07 eV) and OXD (2.98 eV) can efficiently transfer their E_T_ to silylene-diphenylene backbone (referred to 2.67 eV of Si(dBu)), and the TPA-OXD exciplex forms upon photoexcitation in which one component of the pair is originally in the photo-excited state and the other is in the ground state. Thus, it is forbidden for directly energy transfer from the side arm moieties to TPA-OXD exciplex (2.42 eV) since the exciplex exists only under excitation but is dissociative in the ground state^27^,^28^. As the system doped with the green phosphor (2.40 eV), the silylene-diphenylene backbone and TPA-OXD exciplex can transfer their triplet energies to the phosphor. Though the triplet states of TPA-OXD and the green phosphor are close, no back energy transfer from the latter to the former could occur. The reason is that the TPA-OXD exciplex is an excited state species rather than ground state species and can not receive the triplet energy from excited Ir-G, which results in highly efficient exciton harvesting for light emission.

### Phosphorescence PLED device performance

**Figure 3:**
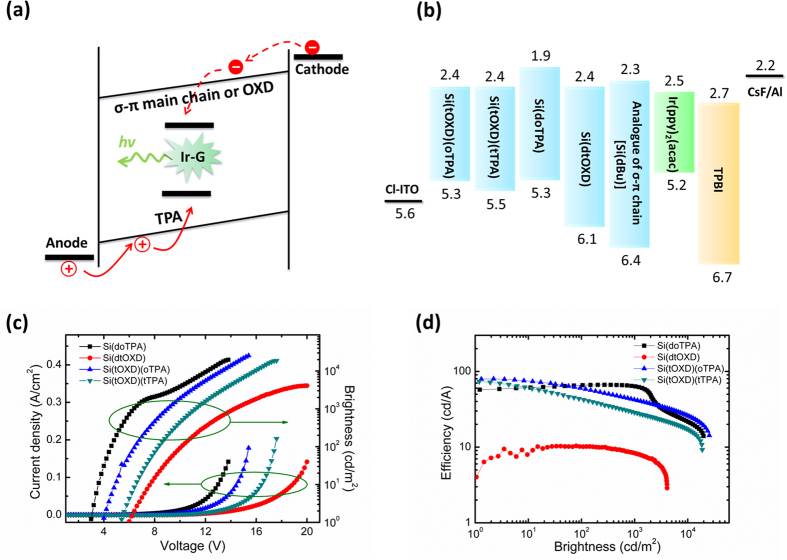
Phosphorescence PLED device and emission mechanisms and performance. Schematic diagram of (**a**) the charge trapping route for the Si(doTPA) and bipolar hosts under electroexcitation and (**b**) the energy level diagram of the materials used. The performance characteristics of (**c**) current density and brightness versus applied voltage and (**d**) luminance efficiency versus brightness of the devices with different Si-diphenylene polymers. The device structure is Cl-ITO/σ-π conjugated polymers: 8 wt% Ir-G (90 nm)/TPBI (65 nm)/CsF (1 nm)/Al.

To obtain highly efficient PLED, it is required to fully utilize singlet and triplet excitons generated in the device as in OLED. This can be done by use of host/guest system with phosphor as dopant and high triplet energy materials as host^29^,^30^. Usually, the host material is so designed such that it can generate high energy singlet and triplet excitons for transferring energy to the guest^2^. In this work, for the bipolar polymer with TPA and OXD, it permits two separated channels for electron and hole that leads to a formation of singlet and triplet excitons in the dopant by charge trapping; for the unipolar polymer Si(doTPA), such charge trapping also occurs but electrons transport via main chain and holes via TPA moiety as shown in Fig. 3a. In addition, the formation of excimer and exciplex allows an additional route for transferring exciton energy to phosphor dopant except for Si(dtOXD) due to its lower excimer E_T_ (2.28 eV) than Ir-G. To simplify the device structure by limiting to the two layers (the host/guest active layer and ETL), we introduce chlorinated indium tin oxide (Cl-ITO) as the anode^19^,^20^ (Fig. 3b), in which the out-layer of ITO is chlorinated for adjusting its work function yet retaining the original high conductivity of the ITO. Here, by appropriate extent of chlorination, Cl-ITO with the work function 5.58 eV was obtained as determined by UPS (Supplementary Fig. 4), which is slightly higher than the HOMO level of TPA 5.3 eV allowing a barrier free for hole injection and an elimination of PEDOT:PSS layer as usually used as HTL in PLED. The matching of the HOMO level of TPA (5.3 eV) with the work function of the Cl-ITO (5.58 eV) as anode and its large gap (0.8 eV) with HOMO of OXD (6.1 eV) assure the hole injection from the anode directly to TPA and transporting among TPA moieties but not to OXD moieties. And the significantly lower difference in LUMO levels of OXD (2.4 eV) with ETL TPBI (2.7 eV) than that of TPA (1.9 eV) with TPBI (0.3 eV vs 0.8 eV), allowing electrons being mainly injected to and transporting among OXD moieties. Both transporting electrons and holes are subsequently trapped into the Ir-G as its HOMO/LUMO levels are laid within HOMO of TPA and LUMO of OXD. Some transporting electrons and holes along OXD and TPA, respectively, could form exciplex in singlet and triplet states, which then transfer their energy to Ir-G. Besides, the LUMO value of Si(dBu) is 2.33 eV, which means the silylene-diphenylene main chain can also provide another channel for electron transport. For the unipolar polymer, Si(doTPA), electrons are mainly injected to main chains, while holes are only injected to TPA moieties and transport across them.

The performance characteristics of the devices with slylene-diphenylene polymers: Cl-ITO/Silylene-diphenylene polymers: 8 wt% Ir(ppy)_2_(acac) (90 nm)/TPBI (65 nm)/CsF (1 nm)/Al are shown in Fig. 3c,d and Table 3. The thickness of the emitting layer is optimal at this 90 nm (Supplementary Fig. 9). For the bipolar host systems, it takes both advantages of efficient charge injections due to no hole injection barrier (from Cl-ITO 5.6 eV to TPA 5.3 eV) and low electron injection barrier of 0.3 eV (from TPBI 2.7 eV to OXD 2.4 eV), and unipolar charge transport along each transport moiety. The optimal performances of the devices with Si(tOXD)(oTPA) and Si(tOXD)(tTPA) are better than those device with unipolar polymers owing to better balanced current densities. For Si(tOXD)(oTPA) device, the maximum brightness and luminescence efficiency are 25452 cd/m^2^ and 80.1 cd/A (corresponding power efficiency 62.9 lm/W and external quantum efficiency 21.2%), respectively. And for Si(tOXD)(tTPA) device, the corresponding values are 18750 cd/m^2^ and 73.5 cd/A (42.7 lm/W and 19.5%). Based on the energy level diagram in Fig. 3b, the charge injections and transport behaviors are revealed below. Since the work function of Cl-ITO is 5.58 eV, there is no barrier for hole injection to the host polymers with the hole transport moiety TPA. For the unipolar host system Si(doTPA) (dual oTPA moieties), though it has no electron transport moiety, its current density profile is higher than those of the two bipolar devices, Si(tOXD)(oTPA) and Si(tOXD)(tTPA). In this device, the electron injection barrier (0.8 eV) from TPBI (2.7 eV) to TPA (1.9 eV) is rather high, thus electrons are mainly injected to the main chains as its LUMO (2.33 eV) is much closer to that of TPBI giving a lower injection barrier of 0.37 eV. The luminescence efficiency increases from 57.4 cd/A (at 1 cd/m^2^) to 66.5 cd/A (at 300 cd/m^2^) and remains steady up to 1000 cd/m^2^ (with 63.3 cd/A) (Fig. 3d). In the practical luminescence operating range: 100–1000 cd/m^2^, the unipolar host system with TPA is significantly better than the bipolar host systems. However, the poor device performance is observed when using Si(dtOXD) as the host, its maximum brightness and efficiency are lower, only 4087 cd/m^2^ and 10.3 cd/A, respectively, due to the large hole injection barrier from Cl-ITO to OXD moiety (0.5 eV) and even higher barrier to the main chain (0.8 eV).

As indicated in the PL spectra of the bipolar polymers (Supplementary Fig. 3), Si(tOXD)(tTPA) and Si(tOXD)(oTPA), the incorporation of tert-butyl group to replace hexyloxy group in TPA can reduce the exciplex formation with OXD due to steric hindrance provided by the bulky tert-butyl group as reflected in the higher fraction of photoexcited exciplex emission 59.2% of the latter than 44.4% of the former (Fig. 2), which is in agreement with the higher EQE of the latter 21.2% than the former 19.5% (Table 3). These results indicate that the triplet energy transfer from the exciplex to the phosphor also play a significant role in addition to charge trapping. It should be mentioned here, although the E_T_ of the exciplex 2.42 eV is very close to that of Ir-G 2.40 eV, the back energy transfer from Ir-G to exciplex would not occur.

### Emission mechanism of phosphorescence PLED device

**Figure 4:**
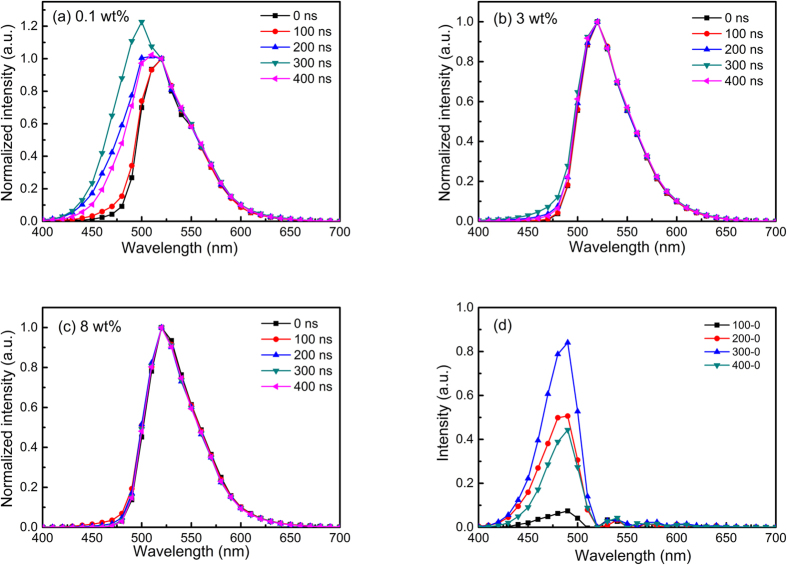
Time Resolved Electroluminescence (TREL) spectra in 0–400 ns. Device: Cl-ITO/Si(tOXD)(oTPA): x% Ir-G (90 nm)/TPBI (65 nm)/CsF/Al with different doping levels of Ir-G: (**a**) 0.1 wt%, (**b**) 3 wt%, and (**c**) 8 wt%. (**d**) The spectra of subtracting 0 ns intensity from 100–400 ns in 0.1 wt% dopant system.

To examine the occurrence of charge trapping, we performed time-resolved electroluminescence (TREL) measurements within 0–400 ns at a narrow voltage pulse (1 MHz, pulse width 300 ns at 10 V) with different Ir-G contents (0.1, 3 and 8 wt%) in Si(tOXD)(oTPA) (Fig. 4); the experimental setup and condition are provided in Supplementary Fig. 10. The extra emission at 490 nm from the host in the case of 0.1 wt% indicates a presence of exciplex singlet emission close to those by photoexcitation 476 nm (Supplementary Table 2). The exciplex emission not only can be observed in the 0.1 wt% dopant system, but also in higher dopant contents (weak at 3 wt% and further weaker or nearly disappeared at 8 wt%). This result supports that the emitting mechanism is mainly by charge trapping of holes and electrons from the host to the guest phosphor dopant, Ir-G, which then form triplet exciton and emit green phosphorescence.

## Conclusion

We have demonstrated that σ-π conjugated polymers with silylene-diphenylene as the backbone repeat unit incorporated with the high triplet energy (3.0 eV) hole transport moiety (triphenyl amine, TPA) and/or electron transport moiety (oxadiazole, OXD) as side arms on silylene exhibit multiple triplet states, including those of main chain as well as transport moieties and molecular complexes between them. The unipolar and bipolar polymer hosts when doped with 8 wt% Ir-G can be used as emitting layer, along with Cl-ITO as anode and TPBI as ETL, to compose of a two-layer-only LED. The device with the polymer host Si(tOXD)(oTPA) exhibits high performance with the maximum luminance efficiency 80.1 cd/A (EQE 21.2%), power efficiency 62.9 lm/W and brightness 25452 cd/m^2^, which is the highest record among the reported phosphorescence green emission PLED. For the unipolar polymer host Si(doTPA), the main chain acts as electron transport channel with low injection barrier and TPA as efficient hole transport channel, which gives high performance with very steady efficiency up to 1000 cd/m^2^. In addition, the present two-layer-only device provides significant lower fabrication cost than the 3~5 layers OLED. Note also that the σ-π conjugated backbone with the silylene linkage for direct incorporation of charge transport moieties without spacer into the polymer as side arms provides a more effective and flexible route for tuning charge injection/transport toward charge balance, yet maintaining higher triplet energy relative to the conventional conjugated polymers. The σ-π conjugated polymer strategy for high triplet energy polymer host is deserved for further investigation for high electro-phosphorescence efficiency in PLED.

## Methods

### Determination of energy levels

The HOMO/LUMO energy levels are determined from CV, absorption spectroscopy and UPS measurements as shown in the Supplementary Information. For Ir(ppy)2(acac) and TPBI, the energy levels are taken from the literatures^31^,^32^.

### Device fabrication

For device preparation, an indium tin oxide (ITO) glass substrate was exposed to oxygen plasma at a power of 50 W and a pressure of 193 mTorr for 5 min, followed by 10 minutes UV treatment in a Pyrex Petri dish with 0.2 ml of o-dichlorobenzene to form chlorinate ITO (Cl-ITO), and finally treated with extra 3 minutes UV ozone. All the silylene-diphenylene polymers with 8 wt% Ir(ppy)_2_(acac) (Ir-G) as the green emission dopant were dissolved in chlorobenzene (CB) to make the polymer solutions, and then used them to prepare the solid film by spin-coating on Cl-ITO. TPBI layer used as electron transporting and hole/exciton blocking layer was deposited on top of the polymer layer by thermal evaporation in a vacuum of 2 × 10^−6^ Torr. (where TPBI is 2,2′,2″-(1,3,5-benzinetriyl)-tris(1-phenyl-1-H-benzimidazole)). Finally, a thin layer of CsF or LiF (about 1 nm) covered with aluminum (100 nm) for bipolar device was deposited in a vacuum thermal evaporator through a shadow mask at a vacuum of 2 × 10^−6^ Torr. The fabrication procedure for single carrier device was similar to that for the bipolar device. The hole dominating device structure was Cl-ITO/Polymer (50 nm)/TPBI (65 nm)/MoO_3_ (15 nm)/Al and the electron dominating device was ITO/Al (55 nm)/Ca (25 nm)/polymer (50 nm)/TPBI (65 nm)/CsF (1 nm)/Al. The active area of device was about 8 mm^2^. I–V characteristics of the devices were measured using a KEITHEY-238 source meter and brightness measured with a TOPCON BM-8 luminance meter. The current efficiency of the device in cd A^−1^ was obtained by dividing brightness by its corresponding current density.

## Additional Information

**How to cite this article**: Chang, Y.-T. *et al*. Bipolar and Unipolar Silylene-Diphenylene σ-π Conjugated Polymer Route for Highly Efficient Electrophosphorescence. *Sci. Rep.* **6**, 38404; doi: 10.1038/srep38404 (2016).

**Publisher's note:** Springer Nature remains neutral with regard to jurisdictional claims in published maps and institutional affiliations.

## Supplementary Material

Supplementary Information

## Figures and Tables

**Table 1:** Comparison of solution-processed green phosphorescent PLEDs.

Polymer host	EQE (%)	CE_max_ (cd/A)	PE_max_ (lm/W)	V_on_ (V)	Ref.
*This work* Si(tOXD)(oTPA)^a^	21.2	80.1	62.9	4.0	—
*Mixed host*					
PVK:OP2^a^	13.6	44.6	—	5.9	9
PVK: OXD-7^b^	—	110.7	29.3	12.0	10

^a^Operated under direct current (D.C.) field.

^b^Operated under alternating current (A.C.) field.

**Table 2:** The phosphorescence characteristics of silylene-diphenylene polymers in diluted toluene solutions.

Materials	Phosphorescence		Molecular Complexes		
	Peaks (nm)	E_T_(eV)^a^	Content	Peaks (nm)^f^	E_T_ (eV)
Si(tOXD)(tTPA)	[405, 424]^d^, 467(sh), 503, 536	[3.07]^d^, 2.66^e^	TPA-OXD exciplex (44.4%)	513, 547	2.42
Si(tOXD)(oTPA)	467(sh), 503, 536	2.66^e^	TPA-OXD exciplex (59.3%)	513, 547	2.42
Si(dtOXD)	[416, 446]^d^, 468, 503, 533(sh)	[2.98]^d^, 2.66^e^	OXD excimer (33.1%)	546	2.28
Si(doTPA)	[405]^d^, 465(sh), 493, 533(sh)	[3.07]^d^, 2.67^e^	TPA excimer (2.2%)	494(sh), 546	2.52
Si(dBu)	465(sh)^c^, 493, 533(sh)	2.67^e^	—	—	—
TPA^b^	405, 422	3.07			
OXD^b^	414, 446, 472	3.00	—	—	—
Biphenyl^b^	428, 467, 493	2.85			

^a^The energy of the first peak of the phosphorescence spectrum is taken as the E_T_.

^b^The phosphorescence data are taken from literatures^18^,^25^,^26^.

^c^sh means shoulder.

^d^The quoted values are contributed from the isolated arm groups.

^e^The triplet energy is determined by the first peak of silylene-diphenylene backbone.

^f^The peaks are assigned by deconvolution of the total phosphorescence spectrum.

**Table 3:** The performance characteristics of the two-layer PLED devices.

Host	V_on_^a^ (V)	L_max_ (cd/m^2^)	η_L_ at L = 100 cd/m^2^ (cd/A)	η_L_ at L = 1000 cd/m^2^ (cd/A)	Max. η_L_, η_P_, and EQE^b^ (cd/A, lm/W, %)
Si(tOXD)(oTPA)	4.0	25452 (at 15.4 V)	58.3 (at 6.6 V)	42.4 (at 9.0 V)	80.1 (at 4.2 V), 62.9, 21.2%
Si(tOXD)(tTPA)	5.4	18750 (at 17.6 V)	42.2 (at 8.4 V)	28.6 (at 11.2 V)	73.5 (at 5.4 V), 42.7, 19.5%
Si(doTPA)	3.0	19847 (at 13.8 V)	65.7 (at 4.6 V)	63.3 (at 6.4 V)	66.5 (at 5.2 V), 60.1, 17.6%
Si(dtOXD)	6.0	4087 (at 20.0 V)	10.1 (at 10.2 V)	8.3 (at 14.4 V)	10.3 (at 9.0 V), 4.4, 2.73%

^a^V_on_ is defined as the voltage at 1 cd/m^2^.

^b^The maximum luminance efficiency and maximum power efficiency are denoted as η_L_ and η_P_, respectively.
