# Supplementary material for: Bipolar and Unipolar Silylene-Diphenylene σ-π Conjugated Polymer Route for Highly Efficient Electrophosphorescence
Source: Sci Rep. 2016 Dec 2;6:38404. doi: 10.1038/srep38404 (PMC5133549; doi:10.1038/srep38404)
Supplement: Supplementary Information [file srep38404-s1.doc]

**Supplementary Information**

**Bipolar and Unipolar Silylene-Diphenylene σ-π Conjugated Polymer Route for Highly Efficient Electrophosphorescence**

*Yao-Tang Chang^1,ǂ^, Sunil Sharma^1, ǂ^, Miao-Ken Hung^1,ǂ^, Yu-Hsuan Lee^1^ & Show-An Chen^1^,*

^1^ Department of Chemical Engineering and Frontier Research Center on Fundamental and Applied Sciences of Matters, National Tsing-Hua University, Hsinchu 30013 Taiwan (ROC). ǂ These authors contributed equally to this work and they should be considered as the first author. Correspondence and requests for materials should be addressed to S.-A. C. (email: sachen@che.nthu.edu.tw)

**Supplementary Figures**


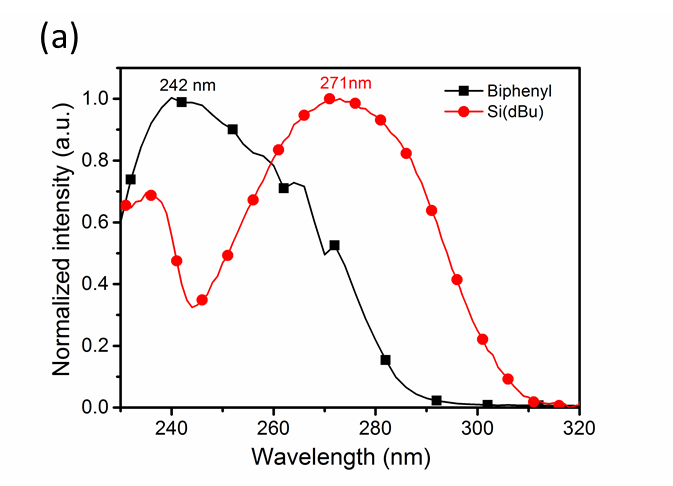

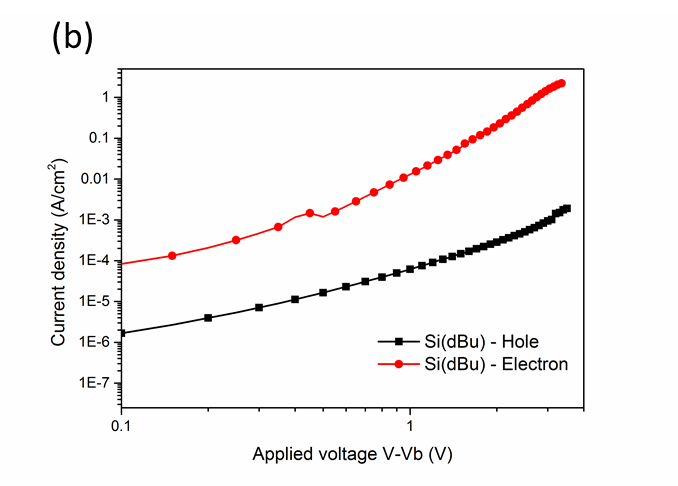


**Supplementary Figure 1 | σ-π conjugated effects.** (**a**) UV-vis absorption spectra in 1× 10^-5^ M chloroform solutions of Biphenyl and Si(dBu). (**b**) Current density versus voltage for single carrier devices with Si(dBu) as the active polymer layer. The hole and electron dominating device structures are ITO/PEDOT:PSS/Si(dBu)/MoO_3_/Al and ITO/Al/Ca/Polymer /CsF/Al, respectively.


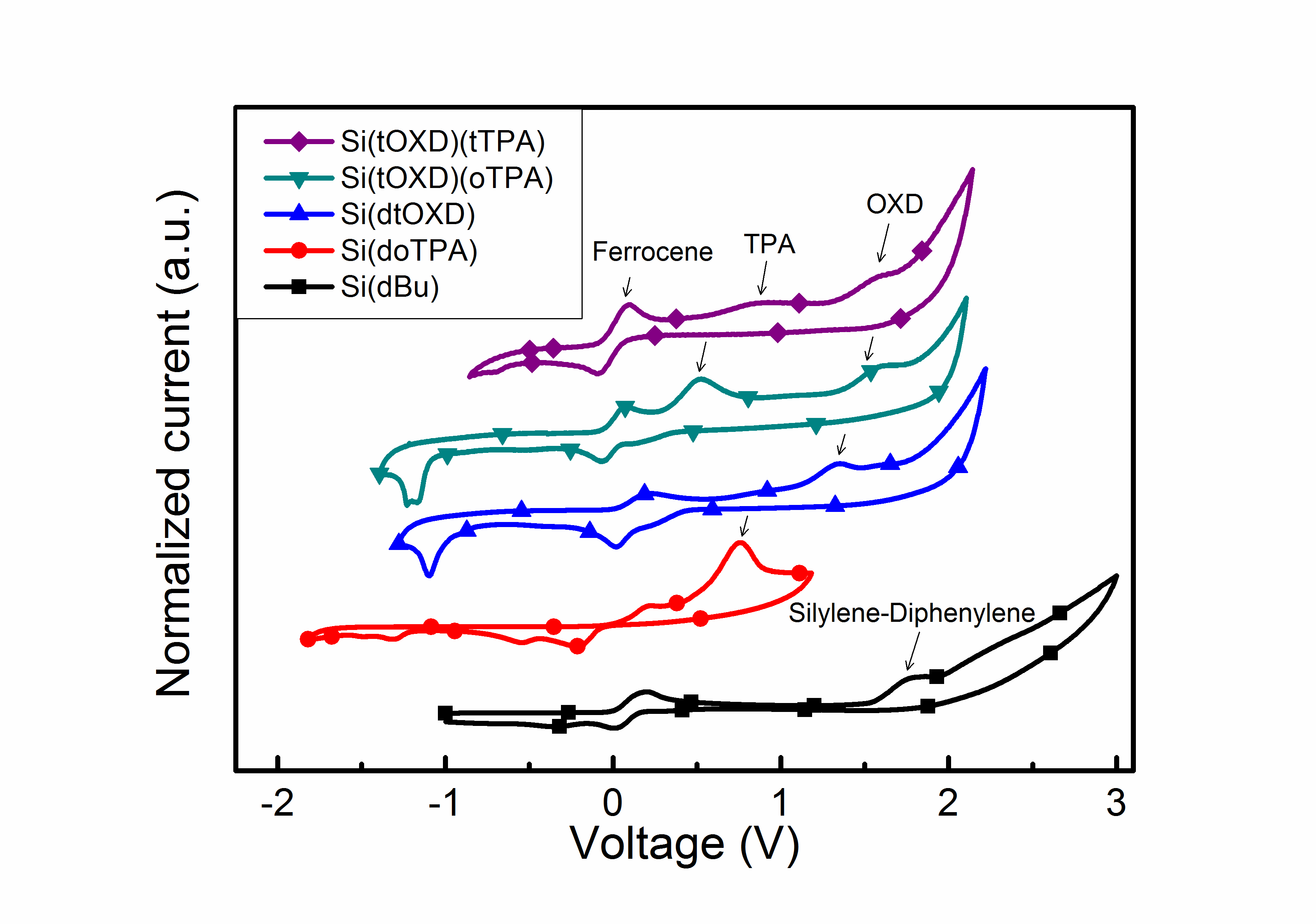


**Supplementary Figure 2 |** Cyclic voltammograms of oxidation for the silylene-diphenylene polymers.


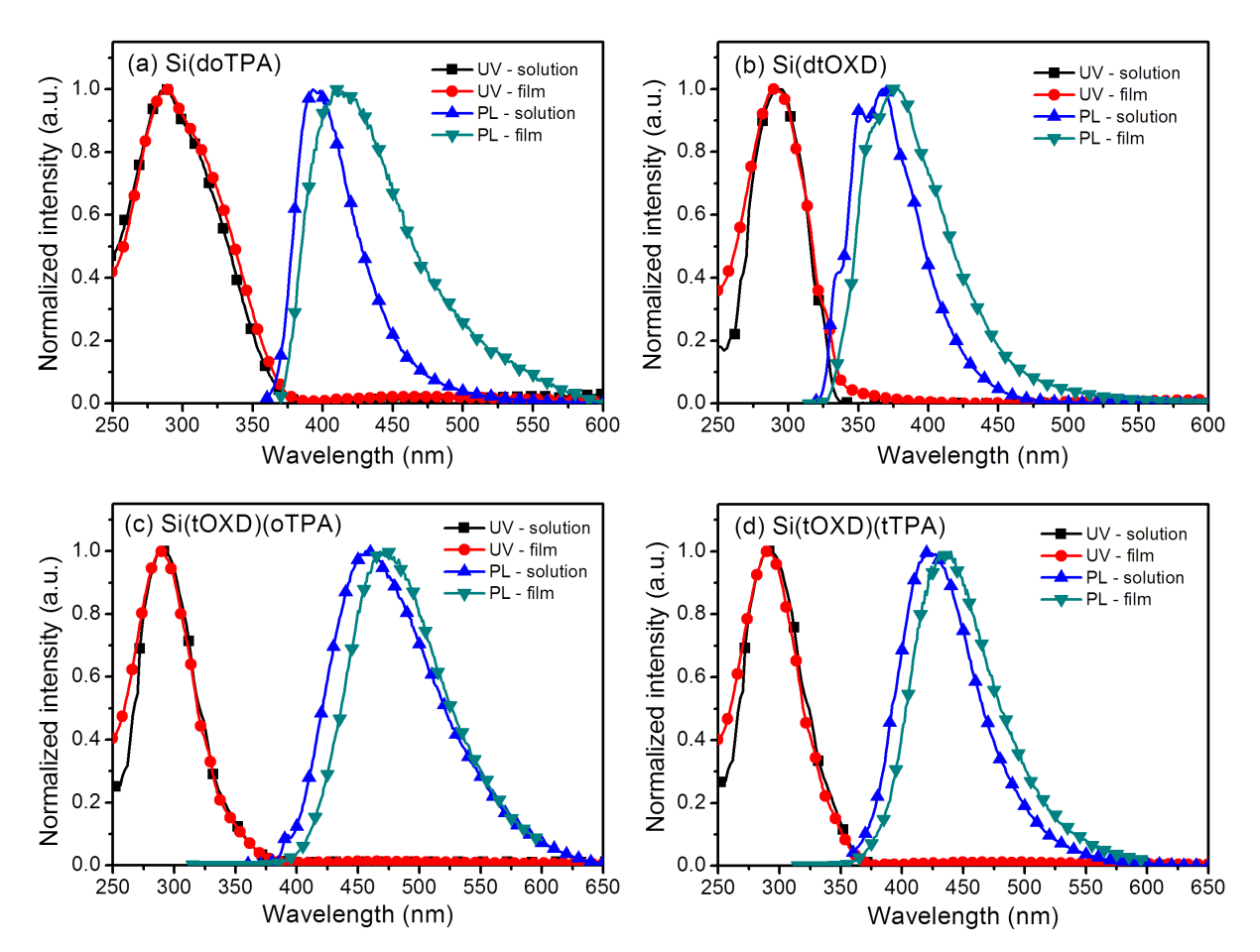


**Supplementary Figure 3 |** **UV-vis absorption and PL spectra in 1× 10^-5^ M chloroform solutions and this solid films.** (a) Si(doTPA), (b) Si(dtOXD), (c) Si(tOXD)(oTPA), and (d) Si(tOXD)(tTPA).


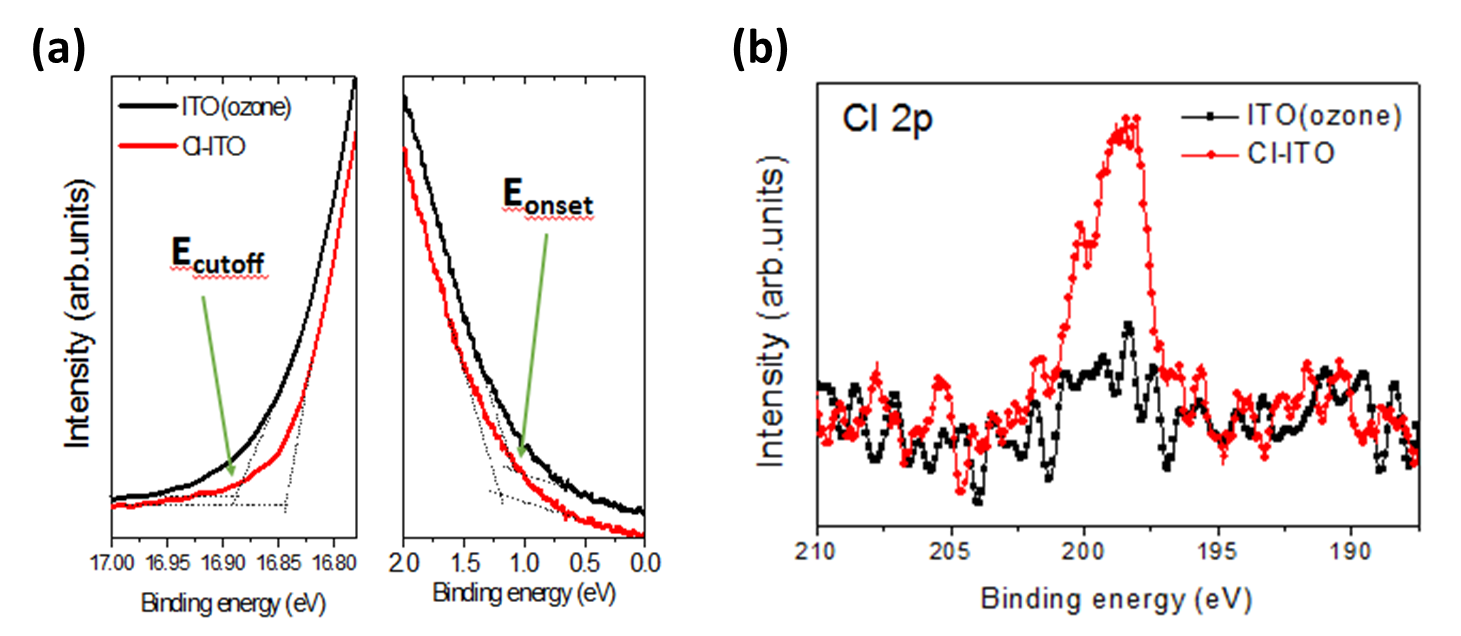


**Supplementary Figure 4 |** Measurement of (a) work function of Cl-ITO by Ultraviolet Photoelectron Spectroscopy and (b) In-Cl bond formation by X-ray Photoelectron Spectroscopy.


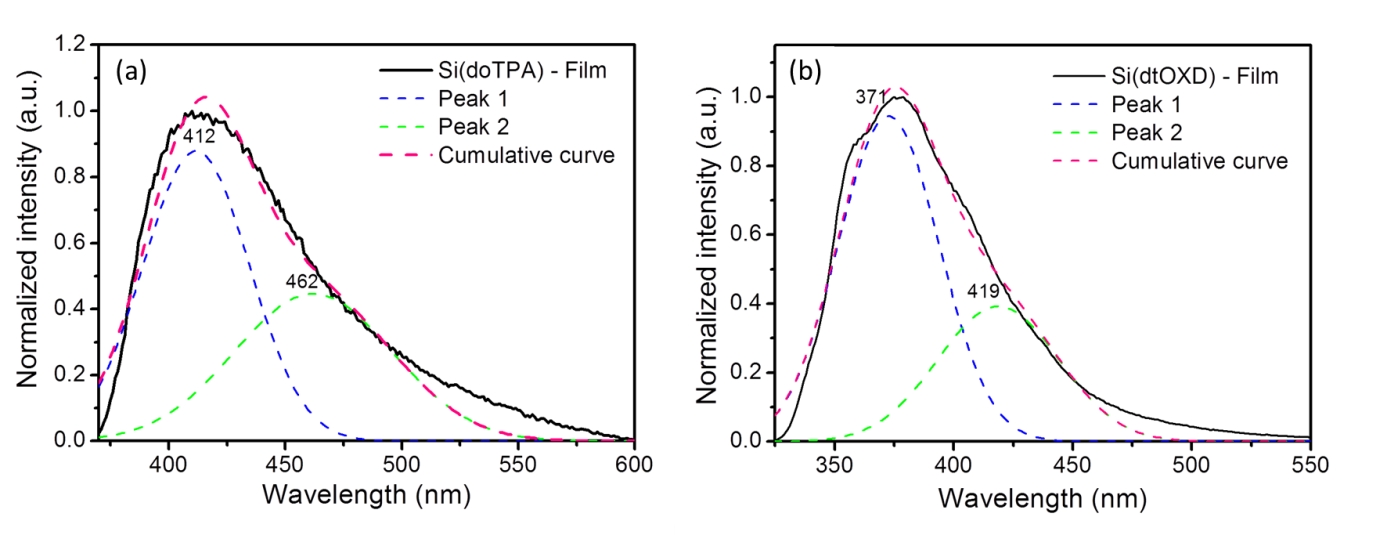


**Supplementary Figure 5 |** The deconvolutions of PL spectra of (a) Si(doTPA) and (b) Si(dtOXD) cast film.

**Supplementary Figure 6 |** PL spectra in thin polymer films and those doped with 8wt% Ir-G of Si(doTPA), Si(dtOXD), Si(tOXD)(oTPA) and Si(tOXD)(tTPA).

**
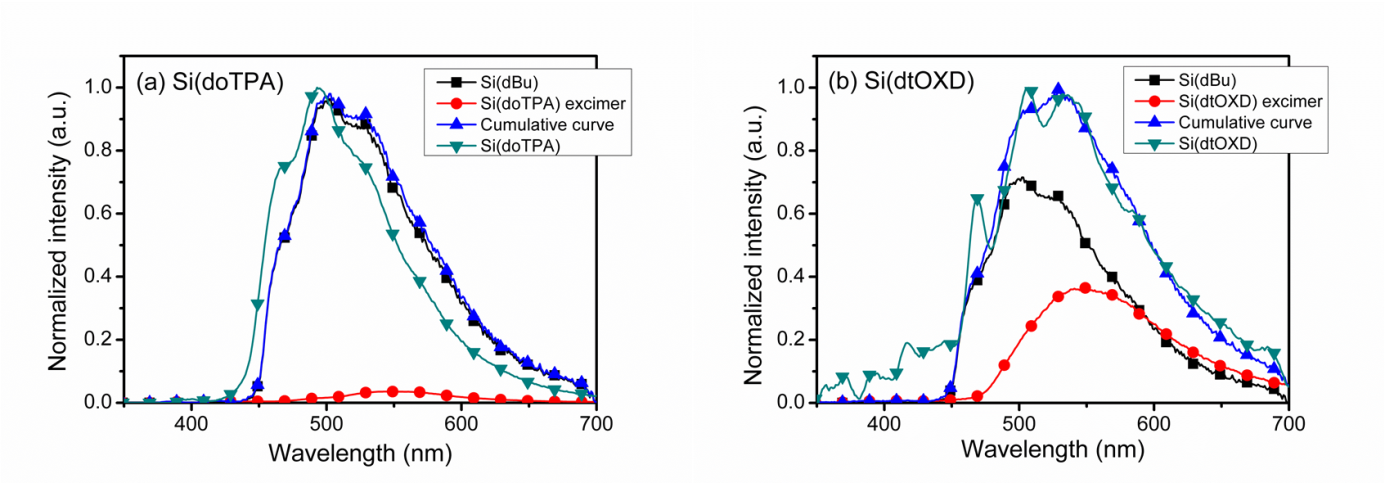
**

**Supplementary Figure 7 |** The deconvolution fitting of phosphorescence spectra for (a) Si(doTPA) and (b) Si(dtOXD) in diluted solution.

**
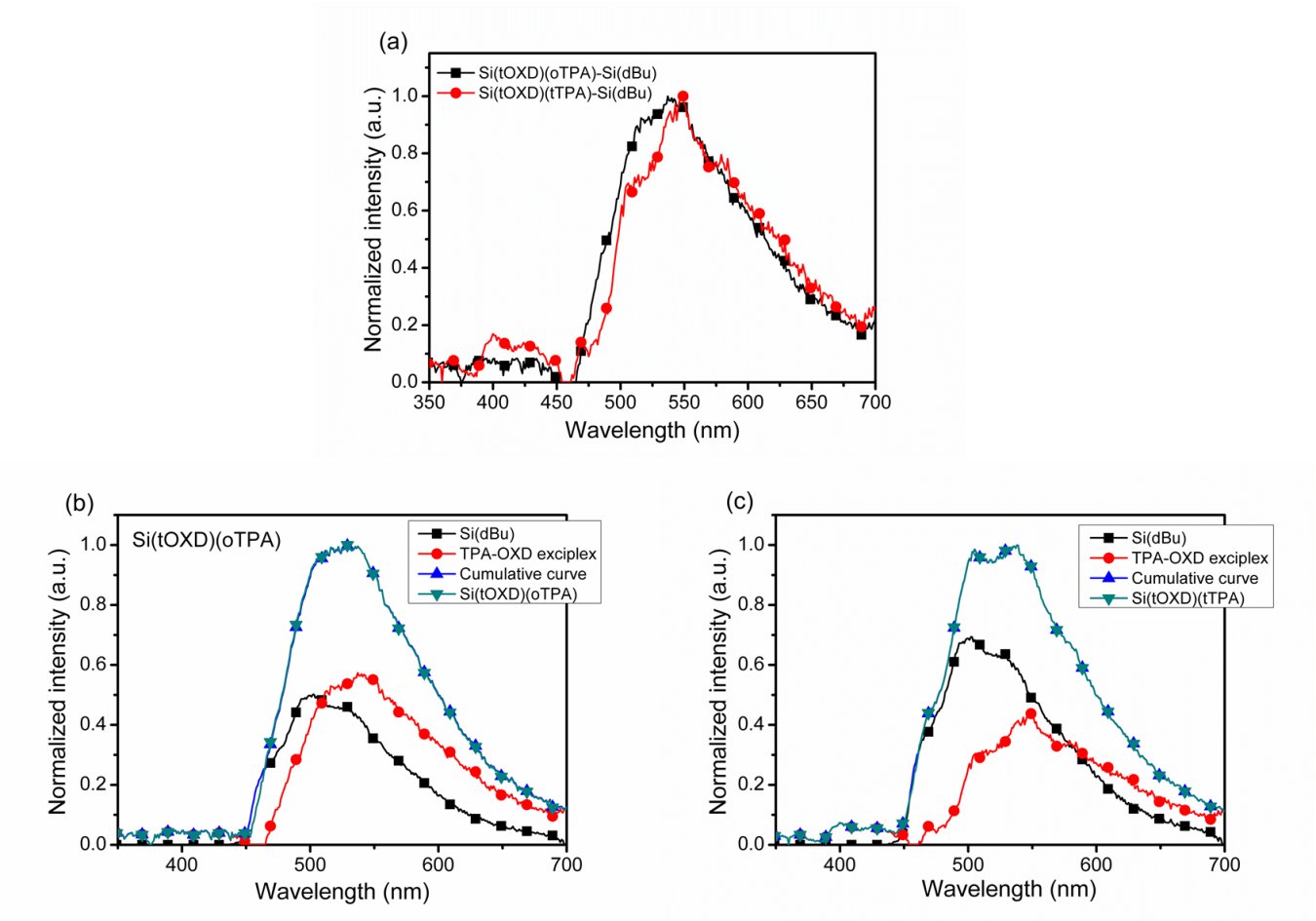
**

**Supplementary Figure 8 |** (a) Phosphorescence profiles by subtracting Si(dBu) spectrum from Si(tOXD)(oTPA) and Si(tOXD)(tTPA) spectra. The deconvolution fitting of Phosphorescence spectra for (b) Si(tOXD)(oTPA) and (c) Si(tOXD)(tTPA).

**
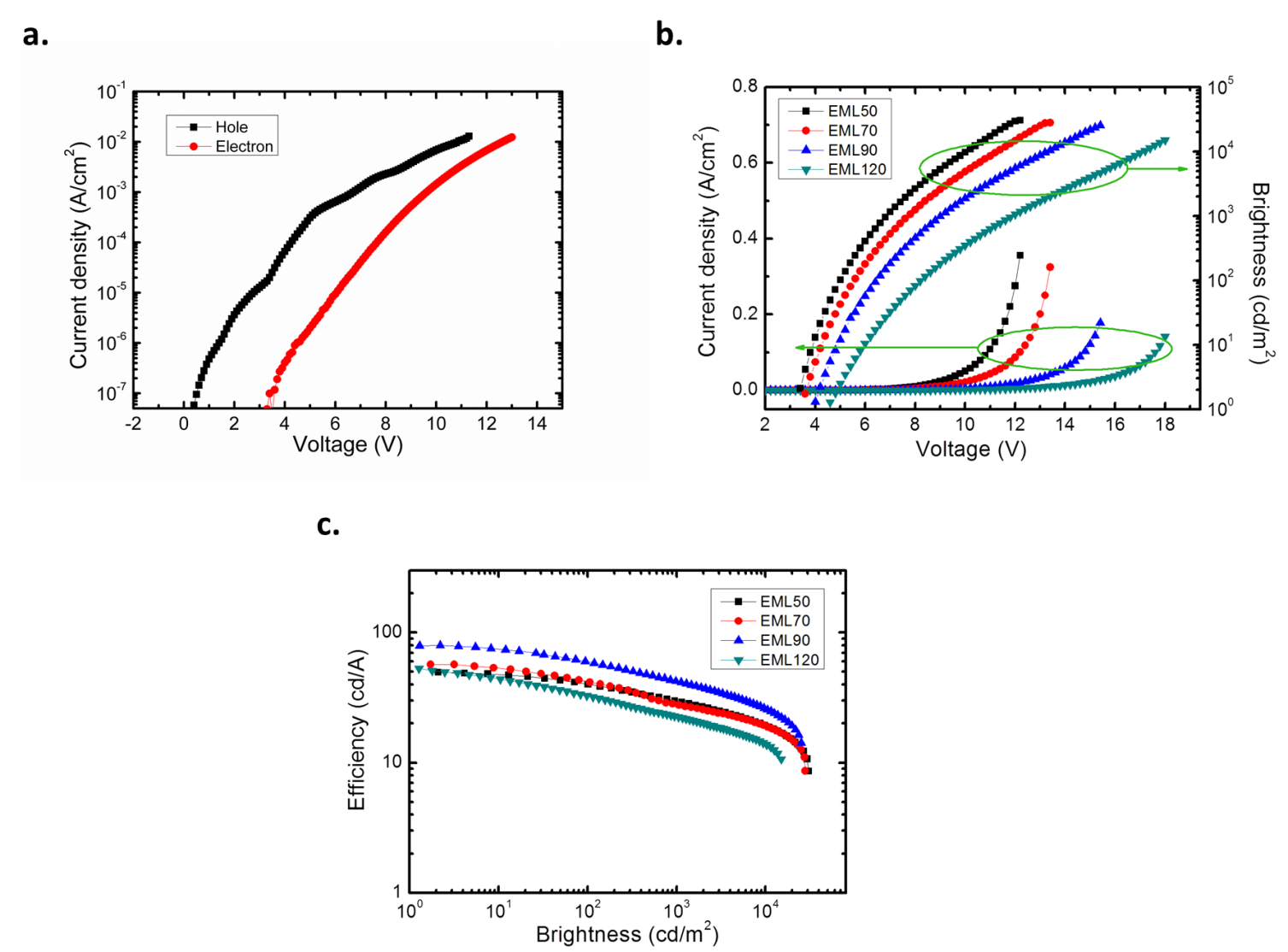
**

**Supplementary Figure 9** **|** **Optimization of the silylene-diphenylene polymer devices.** (**a**) J-V characteristics of the hole-dominated device (square): Cl-ITO/ Si(tOXD)(TPA) (50 nm)/ TPBI (65 nm)/ MoO3 (15 nm)/ Al and the electron-dominated device (circle): ITO/Al (55 nm)/Ca (25 nm)/Si(tOXD)(TPA) (50 nm)/ TPBI (65 nm)/ LiF (1 nm)/ Al. (**b**)(**c**) The performance characteristics with different EML thicknesses of the device: Cl-ITO/ Si(tOXD)(oTPA): 8wt% Ir(ppy)2(acac)/TPBI (65 nm)/CsF (1 nm)/Al.


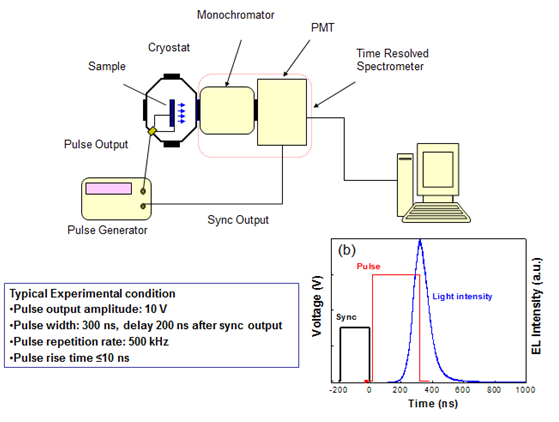


**Supplementary Figure 10 |** Time Resolved Electroluminescence (TREL) measurement. (a) The scheme of TREL instrument setup. (b) The time scale diagram of synchronous trigger, voltage pulse and light emitting spectrum.


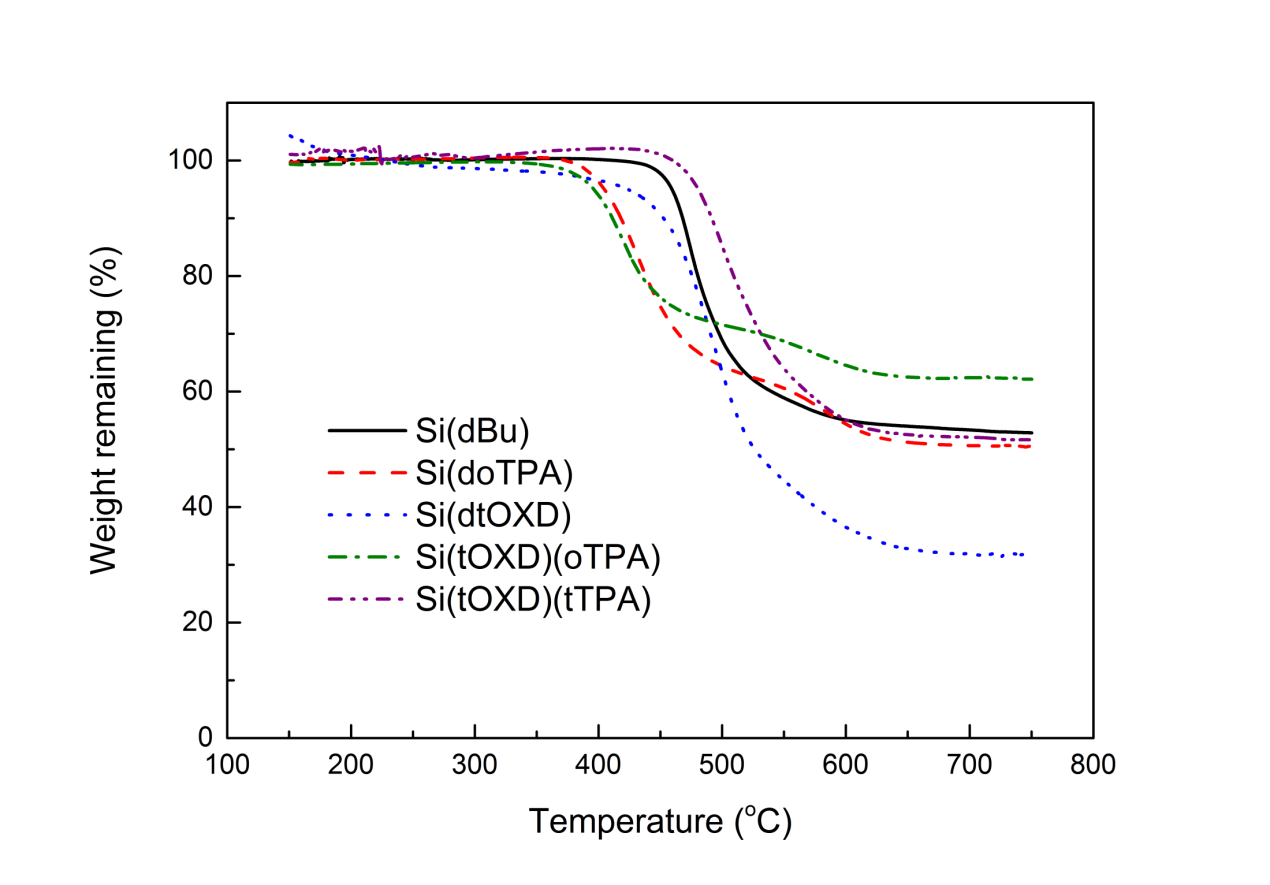


**Supplementary Figure 11** TGA curves of theσ-π conjugated polymers: Si(dBu), Si(doTPA), Si(dtOXD), Si(tOXD)(oTPA), and Si(tOXD)(tTPA).

*
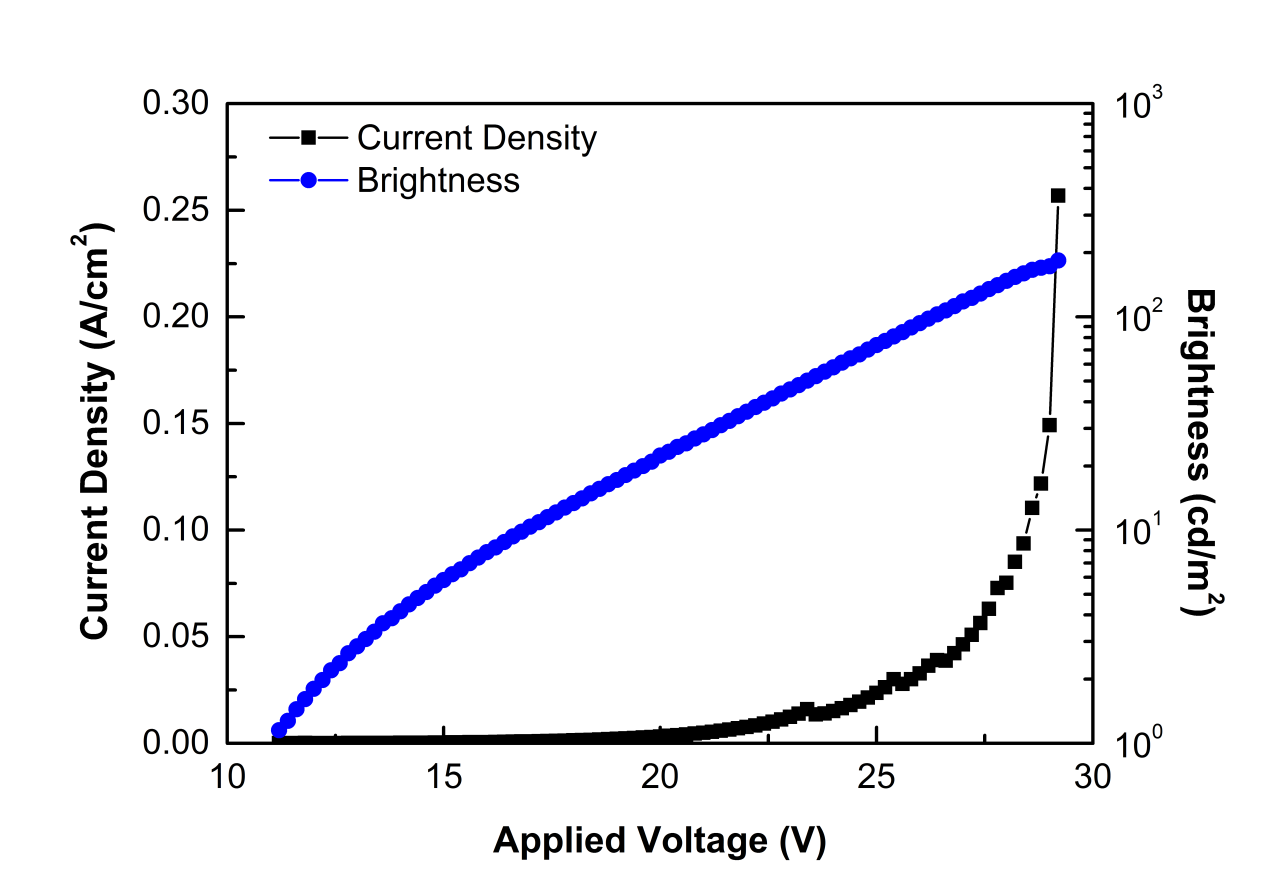
*

**Supplementary Figure 12** The current density and brightness versus applied voltage of the device: Cl-ITO/Si(dBu): 8wt% Ir-G (90 nm)/TPBI (65 nm)/CsF (1 nm)/Al.

**Supplementary Tables**

**Supplementary Table 1 |** HOMO/LUMO levels of silylene-diphenyl polymers.

|  | Oxidation potential vs. Fc/Fc^+^ (V)^a^ | HOMO  (eV) | LUMO^b^ (eV) | Band gap^c^ (eV) |
| --- | --- | --- | --- | --- |
| -2.34  -1.94 | 1.57 | -6.37  -5.34 | -2.31  -1.89 | 4.03  3.40 |
|  | 0.54 |  |  |  |
| -2.37 | 1.3 | -6.10 | -2.70 | 3.73 |
| -2.44 | 0.48, 1.37 | -5.28 (TPA), -6.17 (OXD) | -2.67 | 3.58, (3.73)^d^ |
| -2.42 | 0.71, 1.35 | -5.51 (TPA), -6.15 (OXD) | -2.62 | 3.54, (3.73)^d^ |

^a^Taken as the peak oxidation potential relative to that of Fc/Fc^+^. ^b^E_LUMO_ [eV] = E_HOMO_ + band gap. ^c^Determined from the onset of UV-vis spectrum. ^d^The band gap is taken as that of OXD in Si(dtOXD). Note that the direct measured onset values of UV-vis spectra for the bipolar hosts are contributed from TPA and should not be taken in the calculation of LUMO of OXD.

**Supplementary Table 2 | Absorption and emission peaks of silylene-diphenylene polymers and their corresponding side arm molecules.**

|  | Absorption (λ_max_) | | | Emission (λ_max_) | |
| --- | --- | --- | --- | --- | --- |
| Materials | Solution (nm)^b^ | Film (nm) | Solution (nm)^b^ | | Film (nm) |
| TPA^a^  OXD^a^  Si(doTPA) | 298  283  286 | --  --  286 | | 397  346  393 | --  --  410 |
| Si(dtOXD) | 292 | 292 | | 367 | 377 |
| Si(tOXD)(oTPA) | 290 | 290 | | 456 | 476 |
| Si(tOXD)(tTPA) | 290 | 290 | | 424 | 436 |

^a^The absorption and emission data are taken from the literatures.^2^ ^b^Dissolved in 1× 10^-5^ M chloroform solution; sh means shoulder.

**Supplementary Notes**

**Supplementary Note 1. σ-π conjugated effects.** Supplementary Fig. 1a shows the UV-vis spectra of the dilute solutions (1x10^-5^ M in chloroform) of Biphenyl and Si(dBu). The characteristic absorption peak of Biphenyl is at 242 nm and that of Si(dBu) is at 271 nm; the 29 nm red-shift relative to biphenyl peak indicating a σ-π conjugation between silylene and diphenyl due to extended electron delocalization. In order to determine the electron and hole mobilities of Si(dBu), the current density versus applied voltage for its hole-dominated (ITO/ PEDOT:PSS (30 nm)/Si(dBu) (100 nm)/MoO_3_ (10 nm)/Al) and electron-dominated (ITO/Al (55 nm)/Ca (25 nm)/ Si(dBu) (100 nm)/CsF (1.5 nm)/Al) devices were measured as shown in Supplementary Figure 1b . By applying the space-charge limited current (SCLC) equation, J = 9/8 ε ε_0_ μ V^2^ d^-3^ (where J is the current density, V the bias voltage, μ the carrier, ε the relative vacuum permittivity (about 3 for organic materials), and ε_0_ the vacuum permittivity), its hole mobility of 2.76 × 10^-7^ cm^2^ V^-1^ s^-1^ and electron mobility of 8.47 × 10^-5^ cm^2^ V^-1^ s^-1^ were determined.

**Supplementary Note 2. Electrochemical study.** Supplementary Fig. 2 shows the cyclic voltammograms (CV) of oxidation for the polymers in films on Pt plate as working electrode and Pt as counter electrode in acetonitrile solution of tetra-n-butylammonium tetrafluoroborate (TBABF_4_) as supporting electrolyte (0.1 M) and saturated calomel electrode (SCE) as reference electrode at a scan rate of 100 mV/s. While collecting CV readings, a Pt plate with silylene-diphenylene polymer film was dipped in an electrolyte solution containing little ferrocene (Fc) as the internal standard. According to Leeuw et al,^1^ the ionization potential (E_HOMO_) of a polymer is approximately equal to the peak oxidation potential relative to that of Fc^+^/Fc plus 4.8 eV (the Fc^+^/Fc energy level below the vacuum level), which means that E_HOMO_ can be calculated using IP [eV] = E_onset_ + 4.8 eV. The energy level of LUMO was deduced from the onset of UV-vis spectrum (band gap) and that of HOMO, which means that E_LUMO_ [eV] = E_HOMO_ + band gap.

Their corresponding HOMO/LUMO levels are -6.37/-2.33, -5.34/-1.94, -6.10/-2.37, -5.28/-2.44 and -5.51/-2.42 eV for Si(dBu), Si(doTPA), Si(dtOXD), Si(tOXD)(oTPA) and Si(tOXD)(tTPA), respectively (Supplementary Table 1). Both transport groups possess individual properties. Besides, the higher HOMO values of tTPA than that of oTPA is resulted from hindrance for oxidation provided by the bulky tert-butyl substituent of tTPA.

**Supplementary Note 3. Ultraviolet-visible (UV-Vis) and photoluminescence (PL) spectroscopies.** Supplementary Fig. 3 shows the UV-vis absorption and corresponding PL spectra of thin films and dilute solutions in chloroform (1× 10^-5^ M (based on repeat unit)) of the σ-π polymers: Si(doTPA), Si(dtOXD), Si(tOXD)(oTPA) and Si(tOXD)(tTPA). Each of the corresponding PL spectra was excited by the light at the absorption λ_max_ about 290 nm, and the characteristic values of these spectra are listed in Supplementary Table 2.

Si(doTPA) and Si(dtOXD) exhibit absorption peaks at 286 and 292 nm, respectively, which are 12 nm blue-shift and 9 nm red-shift compared to their side arm corresponding molecules, TPA at 298 nm and OXD at 283 nm.^2^ For the bipolar materials, Si(tOXD)(oTPA) and Si(tOXD)(tTPA), the absorption peaks are both at 290 nm. The emission spectra of the four materials exhibit a similar trend of red-shifts in 10-20 nm from 1× 10^-5^ M dilute solutions to neat films. They show the changes: 393 to 410, 367 to 377, 456 to 476, and 424 to 440 nm from the solutions to the thin solid films for Si(doTPA), Si(dtOXD), Si(tOXD)(oTPA) and Si(tOXD)(tTPA), respectively. Furthermore, the absorption spectra of Si(doTPA) and Si(dtOXD) in the dilute solutions are almost identical to their cast films, but both the emission spectra of the films (Supplementary Fig. 3 a,b) show a broad tail extending to longer wavelength compared to those of the dilute solutions, which reveal that their longer wavelength emission may arise from excited complex species. From a deconvolution of origin peak analyzer software (Supplementary Fig. 5), the Si(doTPA) shows an isolated emission peaked at 412 nm as in the dilute solution, and another emission peak at 462 nm was observed, which reveals a presence of the excited state species, TPA excimer.^4^ Likewise, by deconvoluting the emission spectrum of Si(dtOXD) cast film, a new peak appears at 419 nm, which can be attributed to emission from OXD excimer. For the bipolar polymers Si(tOXD)(oTPA) and Si(tOXD)(tTPA), their emission spectra are significantly red-shifted compared to those of Si(doTPA) and Si(dtOXD), indicating a presence of new species different from that of individual side arms contributions. However, there are no extra absorptions observed in their UV-vis spectra (Supplementary Fig. 3 c,d), which indicates that the emission is probably resulted from an excited complex species ascribed to TPA-OXD exciplex.^3^

**Supplementary Note 4. Measurements of work function of Cl-ITO by Ultraviolet Photoelectron Spectroscopy and of In-Cl bond formation by X-ray Photoelectron Spectroscopy.** We use ultraviolet photoelectron spectroscopy (UPS, from Thermo Electron Corporation) to study surface electronic structure of ITO after ozone and o-dichlorobenzene (ODCB) treatments to give chlorinated ITO (Cl-ITO) as shown in Supplementary Fig. 4. The work functions of ITO (after ozone treatment) and Cl-ITO are 5.0 eV and 5.58 eV, which are higher than that of bare ITO 4.7 eV by 0.3 eV and 0.88 eV, respectively. The work functions are determined by the following equation, work function = *hv* - ( E_cutoff_ - E_onset_), where *hv* is incident photon energy (21.2 eV) of He(I), the high binding energy cutoff (E_cutoff_) and ionization potential (IP) region (E_onset_) are the turning points. The E_cutoff_ is determined by linear extrapolation to zero at the yield of secondary electrons, and the E_onset_ is the onset relative to the Femi level (E_f_) of Au (0 eV), where E_f_ is determined from Au substrate. X-ray photoelectron spectroscopy (XPS) was used to characterize the surface composition of the Cl-ITO electrode. Compared to standard ITO with ozone treatment, the Cl-ITO sample shows the Cl 2p binding energy (BE) peak at 199.06 eV, which verifies the formation of In-Cl_3_ bond (at 199.06 eV).^5^

**Supplementary Note 5. PL spectroscopy of the host polymer films with and without Ir-G dopant.** Supplementary Fig. 6 shows the PL spectra of the four host polymers as films without and with 8wt% Ir-G.Their PL peaks without Ir-G are at 410 nm (Si(doTPA)), 377 nm (Si(dtOXD)), 476 nm (Si(tOXD)(oTPA)) and 436 nm (Si(tOXD)(tTPA)) (Supplementary Table 2). The peaks of the bipolar polymers are significantly red-shifted relative to those of the unipolar polymers. Such red-shifts are probably due to that the bipolar materials contain both electron-rich TPA and electron-deficient OXD moieties, which could mutually interact and form exciplex.^3^ As the four host materials are doped with 8 wt% Ir-G, their PL spectra are almost identical and only exhibit green emission with a peak at 520 nm from Ir-G, indicating that the exciplex transfer its energy to Ir-G completely.

**Supplementary Note 6. Delayed phosphorescence measurement.** The triplet energies of the σ-π conjugated polymers so prepared are determined by taking the maximum of the first vibronic transition (S_0_ ν= 0 ← T_1_ ν= 0) of the phosphorescence spectra from their solutions by using delayed phosphorescence measurement.^6^ In the measurements, the samples were dissolved in toluene (5 × 10^-5^ M), and attached to the plain sample holder in a nitrogen cryostat (Oxford Optistat CF-V). The samples were excited at the absorption maxima of the polymers by 150 fs pulsed Ti:sapphire laser (Spectra-Physics Hurricane) at 10 Hz repetition rate in conjunction with an ultrafast optical parametric amplifier (Quantronix TOPAS). The luminescence from the solution was allowed to pass through a monochromator (Princeton Instruments Acton SpectraPro 2300i) and then to a gated intensified CCD camera (Princeton Instruments PIMAX). The detection window of 20 ms width of the intensified CCD was operated synchronously but delayed by 1 ms with respect to the laser pulse. To increase the signal to noise ratio, each final spectrum was obtained by averaging the accumulated spectra obtained from 150 pulses. The laser intensity was about 7 μJ/pulse. All measurements were carried out in a cryostat at 77 K and under a dynamic vacuum of 10^-7^ Torr.

**Supplementary Note 7. Deconvolution fitting of phosphorescence spectra.** To investigate the phosphorescence emitting species containing in the spectra, we further perform deconvolution fittings with possible species emissions. For Si(doTPA), the common emitting species formation is taken as TPA excimer.^4^ To realize the phosphorescence characteristics of TPA excimer, we introduce the TPA-based molecule, 4,4’-Cyclohexylidenebis [N,N-bis(4-methylphenyl)benzenamine] (TAPC) to form cast film for allowing a presence of plenty of TPA excimer, and its phosphorescence spectrum shows characteristic peaks at 494(sh) and 546 nm. Therefore, the triplet energy is determined as 2.52 eV referred to peak 494 nm. Following, we perform a deconvolution fitting to the phosphorescence of Si(doTPA) by the spectra of Si(dBu) (an analogue of the silylene-diphenylene backbone) and TPA excimer as shown in Supplementary Fig. 7a. The results indicate that the phosphorescence of Si(doTPA) comprises the contributions mainly from silylene-diphenylene backbone and few amount (2.17%) of TPA excimer emissions. Surely, both Si(doTPA) and Si(dBu) spectra are very similar, and the characteristic peaks are all at 465(sh), 493 and 533(sh). For Si(dtOXD), the Si(dtOXD) cast film is introduced (Supplementary Fig. 7b); unlike the diluted solution, the broad spectrum may be arisen from the triplet emission of OXD excimer, which exhibits an E_T_ as 2.28 eV referred to its characteristic peak at 546 nm. Also, we deconvolute the phosphorescence spectrum of the dilute Si(dtOXD) solution by the spectra of Si(dBu) and OXD excimer, and find that 33.14% emission is contributed from OXD excimer.

Supplementary Fig. 8a shows the subtraction profiles by normalizing the spectra of Si(tOXD)(oTPA) and Si(tOXD)(tTPA) at 465 nm to the first peak of Si(dBu). Neglecting the side arm moiety emissions in 390-450 nm, both Si(tOXD)(oTPA)-Si(dBu) and Si(tOXD)(tTPA)-Si(dBu) shows peaks at 513(sh) and 547 nm. The two profiles are very similar, which reveals that they are from the same species of TPA-OXD exciplex; and the first shoulder peak 513 nm is determined as 2.42 eV of its triplet state. Furthermore, by deconvoluting the phosphorescence spectra of bipolar polymers as shown in Supplementary Fig. 8b&c, both Si(tOXD)(oTPA) and Si(tOXD)(tTPA) possess considerable amount of TPA-OXD exciplex emissions, which are 59.27% and 44.44% of Si(tOXD)(oTPA) and Si(tOXD)(tTPA), respectively. Since the tert-butyl moiety of the end of TPA (tTPA) possessing much more steric hindrance than the hexyloxy moiety of TPA (oTPA), which probably restricts the structure orientation to form TPA-OXD exciplex, so that Si(tOXD)(oTPA) gives larger amount of exciplex than Si(tOXD)(tTPA).

**Supplementary Note 8. Determination of optimal emitting layer thickness of the silylene- diphenylene polymer devices.** To investigate the voltage dependences of hole and electron current densities in the bipolar device with Si(tOXD)(oTPA) as host and TPBI as ETL, we measured hole and electron current densities in the single carrier devices under the condition: the film thicknesses of EML and ETL are 50 and 65 nm, respectively. The hole dominated device structure is: Cl-ITO/ Si(tOXD)(oTPA) (50 nm)/TPBI (65 nm)/MoO_3_ (15 nm)/Al and the electron dominated device structure is: ITO/Al (55 nm)/Ca (25 nm)/ Si(tOXD)(oTPA) (50 nm)/TPBI (65 nm)/LiF (1 nm)/Al. As shown in Supplementary Fig. 9a, holes start injecting from Cl-ITO at extremely low voltage (< 1 V), but electrons start injecting at 3.4 V indicating that the device with this host polymer and ETL should be turned on at this voltage as it is in fact just the case. In addition, the hole current density is higher than electron current density by two order of magnitude. Due to much more holes than electrons are injected as compared under same electric field, the recombination zone could be very close to the interface of EML/TPBI and even in the region of ETL (TPBI) resulting in that some portions of emitter in EML are not utilized and then in a drop in efficiency. Therefore, we expect an increase in efficiency by increasing the EML thickness along with the use of CsF to replace LiF to promote electron injection. The corresponding performance results of the devices with ETL at 65 nm thick and EML at various thicknesses from 50 to 90 nm are shown in Supplementary Fig. 9b, c. As the thickness of EML increases from 50 to 90 nm, the efficiency increases, but the current density decreases and turn-on voltage increases, while the brightness remains at the same level at about 25000-30000 cd/m^2^. As the thickness further increases to 120 nm, the current density, brightness, and efficiency all drop significantly.

Thus the optimal thickness of EML is 90 nm, at which the device performance achieves the maximum brightness 25452 cd/m^2^, maximum luminance efficiency 80.1 cd/A, maximum power efficiency 62.9 lm/W, and EQE 21.2%. At practical brightness of 100 cd/m^2^, the device exhibits luminance efficiency 60.2 cd/A, power efficiency 29.3 lm/W, and EQE 15.9%. Therefore, it is clear that increase of EML thickness could shift the recombination zone far from the anode (where triplet exciton could be quenched) yet close to EML/TPBI interface and consequently improve the device efficiency. In the meantime, increasing EML thickness also causes the operation voltage higher and results in a drop of power efficiency.

**Supplementary Note 9. Time Resolved Electroluminescence (TREL) measurement.** The experimental setup and condition are similar to our previous report^7^ are shown in Supplementary Fig. 10. Normalized EL spectral dynamics at 520 nm, the first 300 ns period corresponds to the electro-excitation pulse.

**Supplementary Note 10. Thermal stability measurement.** The thermal stabilities of silylene-diphenylene polymers measured by thermogravimetric analysis (TGA) are shown in Supplementary Fig. 11. The TGA measurement reveals that the silylene-diphenylene polymers Si(dBu), Si(doTPA), Si(dtOXD), Si(tOXD)(oTPA), and Si(tOXD)(tTPA) having the thermal decomposition temperature (T_d_) at 5% weight-loss temperature are 459, 405, 424, 396, and 481℃, which indicate that all the silylene-diphenylene polymers have good thermal stability. Besides, we also observe that the polymers having side arms with tert-butyl group have higher T_d_ compared to the polymers having side arms with hexyloxy group.

**Supplementary Note 11. Device performance for the silylene-diphenylene polymer.** Supplementary Fig. 12 shows the performance characteristics of current density and brightness versus voltage of the device: Cl-ITO/Si(dBu): 8wt% Ir(ppy)_2_(acac) (90 nm)/TPBI (65 nm)/CsF (1 nm)/Al. Since the HOMO level of Si(dBu) is 6.4 eV, holes are difficult to inject from Cl-ITO due to the high barrier 0.8 eV that leads to the high turn on voltage (>11 V) and low brightness (184 cd/m^2^).

**Supplementary Methods**

**Materials.** Tetrachlorosilane, 4-bromophenol, and 1,4-dibromobenzene from Alfa-Aesar (99.0% purity), *n*-butyllithium from Chemetall Taiwan Co. Ltd, Triisopropyl borate from Acoss Organics, and 4-(hexyloxy)aniline from Sigma-Aldrich (99.0% purity) were purchased. Tetrahydrofuran (THF) and diethyl ether were dried by refluxing over sodium metal with the indicator benzophenone complex. All other reagents were purchased from commercial suppliers and used as received. Thin-layer chromatography (TLC) and column chromatography were performed on silica gel. Anhydrous toluene and DMF (stored over molecular sieves) were used.

**General measurement and characterization.** ^1^HNMR and ^13^CNMR spectra were recorded on a VARIAN UNITYINOVA 500 NMR in the NSC Regional Instrument Centre at National Tsing Hua University, Taiwan. FAB Mass spectra were also collected using MICROMASS TRIO-2000 in the NSC Regional Instrument Centre at National Chiao Tung University, Taiwan. The thickness of the active layer film, hole transport layer, and interlayer were measured by a Tencor P-10 Surface Profiler. Gel permeation chromatogrphy (GPC) (from Waters) assembled with a UV detector and three columns in series was used to measure molecular weight distributions relative to polystyrene standards at 40°C. Melting points were recorded on a BÜCHI Melting Point B-540. Infrared spectra were recorded on an ATI Mattson Genesis Series FT-Infrared spectrophotometer.


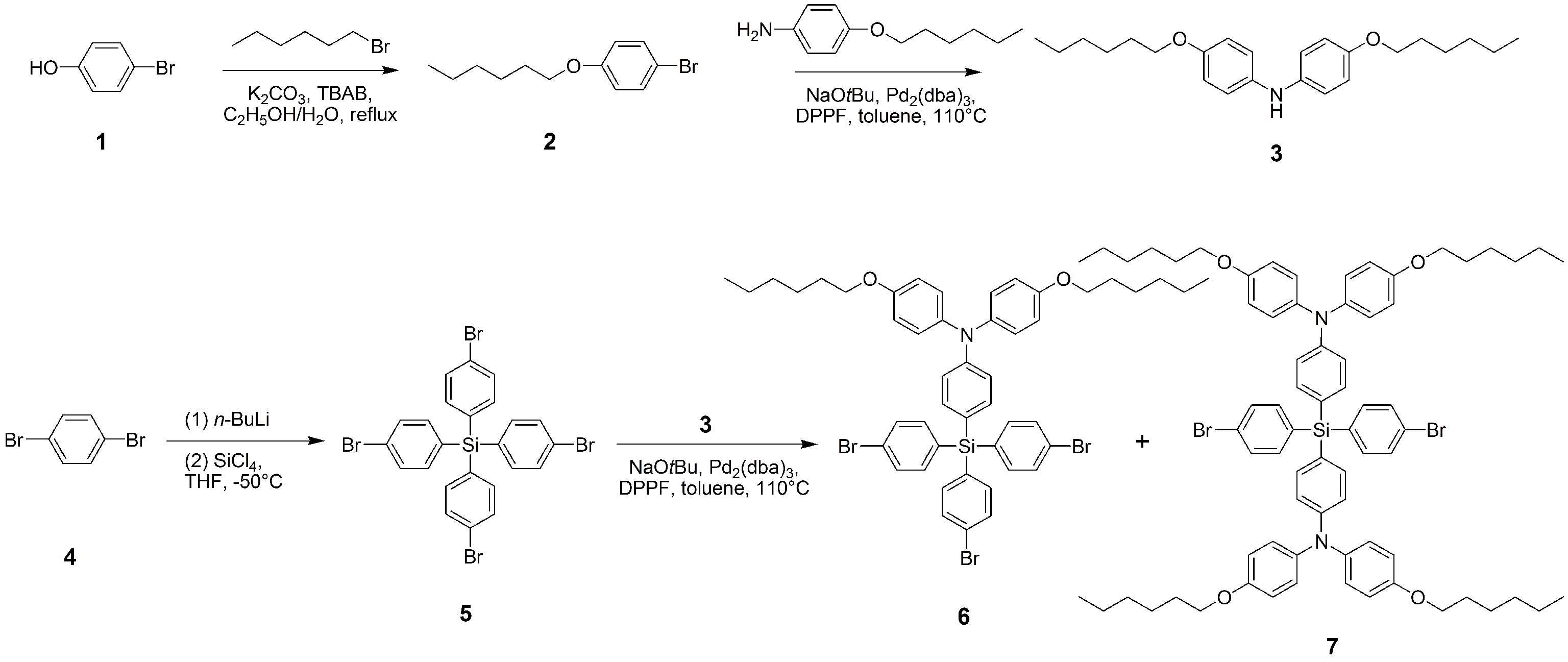


**Supplementary Scheme 1 |****Synthetic route for monomer 7.**

**Synthesis of 1-bromo-4-(hexyloxy)benzene (2).** A mixture of 4-bromophenol **1** (20.0 g, 115.0 mmol), 1-bromohexane (37.29 g, 253.0 mmol), K_2_CO_3_ (47.68 g, 345 .0 mmol) and tetrabutylammonium bromide (0.2 g) in ethanol (300 mL) and H_2_O (100 mL) was refluxed for 12 h. After the completion of the reaction, the reaction mixture was allowed to cool, and solvent was removed under reduced pressure. The residue was dissolved in ethyl acetate (150 mL), and ethyl acetate was washed with water (40 mL), brine (40 mL), and dried over MgSO_4_. The solvent was evaporated under reduced pressure to afford product **2** (29.7 g, 94.3 %) as a Colorless oil. ^1^HNMR (500 MHz, CDCl_3_). δ (ppm): 7.33 (d, *J*= 9.0 Hz, 2H), 6.75 (d, *J*= 9.0 Hz, 2H), 3.86 (t, *J*= 6.4 Hz, 2H), 1.77-1.71 (m, 2H), 1.45-1.39 (m, 2H), 1.33-1.29 (m, 4H), 0.88 (t, *J*= 7.0 Hz, 3H); ^13^CNMR (125 MHz, CDCl_3_). δ (ppm): 158.2, 132.1, 116.2, 112.5, 68.2, 31.5, 29.1, 25.6, 22.5, 14.0; FTIR (Neat) 3032, 2930, 2859, 1590, 1504, 1240, 1067, 733 cm^-1^.

**Synthesis of bis(4-(hexyloxy)phenyl)amine (3).** A mixture of 4-(hexyloxy)aniline (12.0 g, 62.08 mmol), and 1-bromo-4-(hexyloxy)benzene **2** (17.5 g, 68.30 mmol) in toluene (120 mL) was purged with nitrogen for 40 min. Then, sodium *tert*-butoxide (8.94 g, 93.12 mmol), Pd_2_(dba)_3_ (0.567 g, 0.62 mmol), and 1,1'-bis(diphenylphosphino)ferrocene (0.688 g, 1.24 mmol) were added to the mixture and purged with nitrogen for 10 min. The reaction mixture was heated at 110 °C for 2 h under nitrogen atmosphere. The brown suspension was then allowed to cool to room temperature, after which water (50 mL) was added and the mixture was extracted with ethyl acetate (150 mL). The organic solvent was washed with water (50 mL), brine (50 mL), dried over MgSO_4_, and the solvent was removed under reduced pressure. The residue was purified by column chromatography on silica gel, eluting with hexane/ ethyl acetate 97:3, to yield product **3** (19.1 g, 83.4 %) as a pale yellow solid. mp: 75-77 °C (lit.^8^ 77-79 °C); GC-MS calculated C_24_H_35_NO_2_: m/z = 369 Found : m/z 369 (M^+^). ^1^HNMR (500 MHz, DMSO). δ (ppm): 7.47 (br s, 1H), 6.89-6.86 (m, 4H), 6.79-6.77 (m, 4H), 3.86 (t, *J*= 6.2 Hz, 4H), 1.68-1.63 (m, 4H), 1.44-1.36 (m, 4H), 1.31-1.27 (m, 8H), 0.87 (t, *J*= 7.0 Hz, 6H); ^13^CNMR (125 MHz, CDCl_3_). δ (ppm):153.7, 137.8, 119.5, 115.4, 68.5, 31.6, 29.3, 25.7, 22.6, 14.0; FTIR (Neat) 3380, 2956, 2938, 2874, 2864, 1515, 1254, 1030, 830, 797 cm^-1^.

**Synthesis of tetrakis(4-bromophenyl)silane (5)**. Tetrakis(4- bromophenyl)silane **5** was prepared according to a procedure of the literature.^9^. For Compound **5:** A white solid (16 g, 93.0%). mp: 241-243 °C (lit.^9^ 242-243 °C);^1^HNMR (500 MHz, CDCl_3_). δ (ppm): 7.52 (d, *J*= 8.5 Hz, 8H), 7.32 (d, *J*= 7.5 Hz, 8H). ^13^CNMR (125 MHz, CDCl_3_). δ (ppm):137.6, 131.4, 131.4, 125.4; FTIR (Neat) 2963, 1570, 1478, 1066, 1010, 809, 731 cm^-1^.

**Synthesis of 4-(hexyloxy)-N-(4-(hexyloxy)phenyl)-N-(4-(tris(4-bromophenyl)silyl)phenyl) benzenamine (6)**. Compound **6** and **7** were prepared in a similar process to that of **3** but bis(4-(hexyloxy)- phenyl)amine **3** and **5** were used as the starting materials. The products were purified by column chromatography on silica gel, eluting with hexane/ dichloromethane 9:1, to yield pure compound **6** (19.1 g, 62.6 %) as a colorless sticky and with hexane/ dichloromethane 4:2, to yield pure compound **7** (6.5 g, 16.3 %) as a white solid. For Compound **6:** FAB MS calculated C_48_H_50_Br_3_NO_2_Si: m/z = 940.7 Found : m/z 940 (M^+^). ^1^HNMR (500 MHz, CDCl_3_). δ (ppm): 7.48 (d, *J*= 8.5 Hz, 6H), 7.34 (d, *J*= 8.5 Hz, 6H), 7.19 (d, *J*= 7.0 Hz, 2H), 7.05 (d, *J*= 8.0 Hz, 4H), 6.81-6.80 (m, 6H), 3.90 (br s, 4H), 1.77-1.71 (m, 4H), 1.46-1.40 (m, 4H), 1.32-1.30 (m, 8H), 0.88 (t, *J*= 7.0 Hz, 6H); ^13^CNMR (125 MHz, CDCl_3_). δ (ppm):156.1, 150.5, 139.7, 137.7, 136.9, 132.8, 131.2, 127.5, 124.9, 120.5, 118.0, 115.4, 68.2, 31.6, 29.2, 25.7, 22.6, 14.0; FTIR (Neat) 3012, 2947, 1571, 1504, 1467, 1068, 1011, 757, 731 cm^-1^.

**Synthesis of N-(4-((4-(bis(4-(hexyloxy)phenyl)amino)phenyl)bis(4-bromophenyl)silyl) phenyl)-4-(hexyloxy)-N-(4-(hexyloxy)phenyl)benzenamine (7).** For Compound **7:** FAB MS calculated C_72_H_84_Br_2_N_2_O_4_Si: m/z = 1229 Found : m/z 1229 (M^+^). mp: 112-115 °C. ^1^HNMR (500 MHz, DMF-d_7_). δ (ppm): 7.67 (d, *J*= 8.5 Hz, 4H), 7.48 (d, *J*= 8.5 Hz, 4H), 7.33 (d, *J*= 9.0 Hz, 4H), 7.13 (d, *J*= 9.0 Hz, 8H), 6.98 (d, *J*= 9.0 Hz, 8H), 6.83 (d, *J*= 8.0 Hz, 4H), 3.98 (t, *J*= 6.2 Hz, 8H), 1.77-1.71 (m, 8H), 1.46-1.42 (m, 8H), 1.38-1.31 (m, 16H), 0.88 (t, *J*= 7.2 Hz, 12H); ^13^CNMR (125 MHz, CDCl_3_). δ (ppm):155.9, 150.1, 139.9, 137.8, 136.9, 134.1, 130.9, 127.2, 124.4, 122.3, 118.2, 115.3, 68.2, 31.6, 29.3, 25.7, 22.6, 14.0; FTIR (Neat) 2934, 2860, 1591, 1504, 1474, 1238, 1113, 1011, 824, 731 cm^-1^.


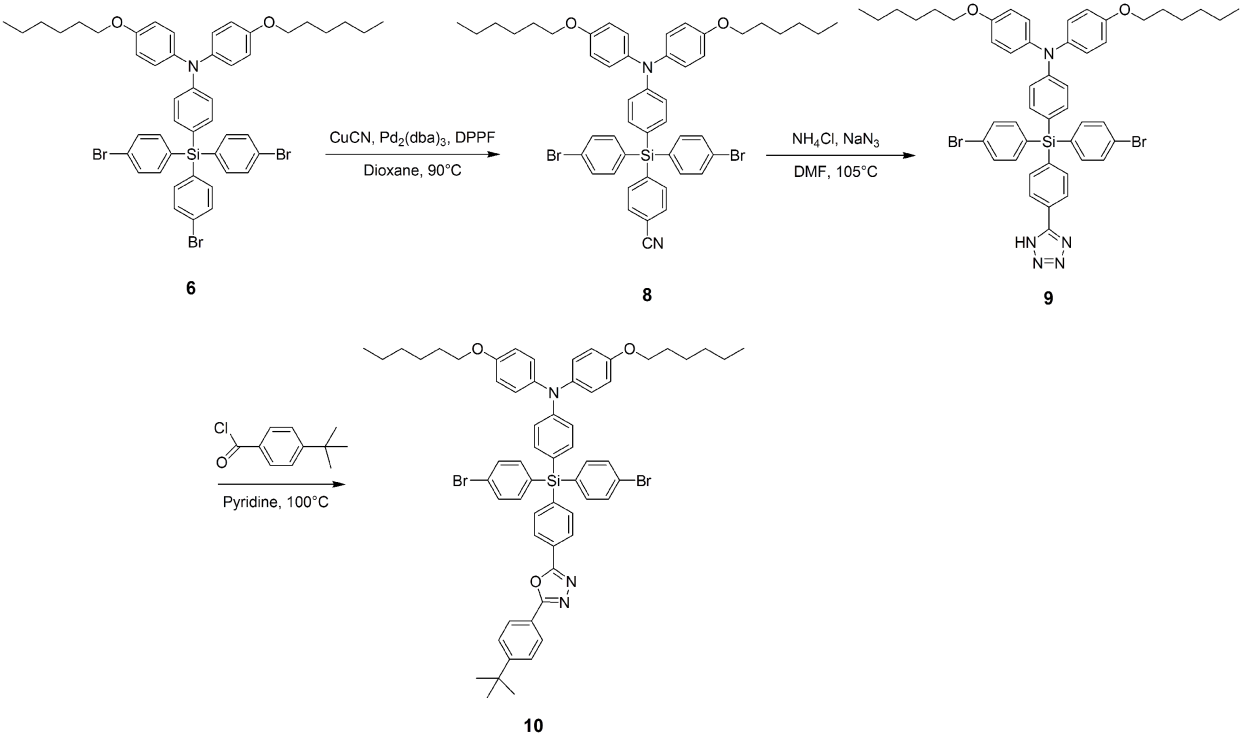


**Supplementary Scheme 2 | Synthetic route for monomer 10.**

**Synthesis of 4-((4-(bis(4-(hexyloxy)phenyl)amino)phenyl)bis(4-bromophenyl)silyl)benzo -nitrile (8)**. A solution of 4-(hexyloxy)-N-(4-(hexyloxy)phenyl)-N-(4- (tris(4-bromophenyl) silyl)-phenyl)aniline **6** (5.0 g, 5.31 mmol) in dioxane (75 mL) was purged with nitrogen for 20 min. Then, CuCN (0.95 g, 10**.**62 mmol) Pd_2_(dba)_3_ (0.092 g, 0**.**106 mmol), and 1,1'-bis(diphenylphosphino)ferrocene (0.253 g, 0**.**425 mmol) were added to the mixture and purged with nitrigen for 5 min. The reaction mixture was heated at 90 °C for 24 h under nnitrogen atmosphere. The reaction mixture was then allowed to cool to room temperature, and the resulting dark brown solution was pass through a pad of Celite, which was washed with ethyl acetate (2 x 40 mL). Finally, the organic solvents were combined, dried over anhydrous MgSO_4_, filtered, and concentrated under reduced pressure. The residue was purified by silica gel column chromatography using hexane and ethyl acetate (97:3) as eluent to afford the title compound **8** (1.9 g, 40 %) as a white solid. 1.5 g of 4-(hexyloxy)-N-(4-(hexyloxy)phenyl)-N-(4- (tris(4-bromophenyl)silyl)- phenyl)aniline **6** was also recovered. mp: 82-85 °C. Analytical data of **8**: ^1^HNMR (500 MHz, CDCl_3_). δ (ppm): 7.62-7.58 (m, 4H), 7.50 (d, *J*= 8.0 Hz, 4H), 7.34 (d, *J*= 8.0 Hz, 4H), 7.17 (d, *J*= 9.0 Hz, 2H), 7.07 (d, *J*= 8.5 Hz, 4H), 6.84-6.80 (m, 6H), 3.90 (t, *J*= 6.5 Hz, 4H), 1.77-1.72 (m, 4H), 1.46-1.40 (m, 4H), 1.33-1.30 (m, 8H), 0.88 (t, *J*= 7.2 Hz, 6H); ^13^CNMR (125 MHz, CDCl_3_). δ (ppm): 156.2, 150.8, 141.1, 139.5, 137.6, 136.9, 136.5, 131.9, 131.4, 131.1, 127.5, 125.2, 119.3, 118.7, 117.9, 115.4, 113.4, 68.2, 31.6, 29.3, 25.7, 22.6, 14.0; FTIR (Neat) 2929, 2858, 2230, 1589, 1504, 1479, 1239, 1112, 1066, 1010, 823, 810, 733 cm^-1^.

**Synthesis of 4-((4-(1H-tetrazol-5-yl)phenyl)bis(4-bromophenyl)silyl)-N,N-bis(4-(hexyl- oxy)phenyl)benzenamine (9).**4-((4-(Bis(4-(hexyloxy)phenyl)amino)phenyl)bis(4-bromo- phenyl)silyl)benzonitrile **8** (1.5 g, 1.69 mmol), sodium azide (0.549 g, 8.45 mmol) and ammonium chloride (0.452 g, 8.45 mmol) were combined in a flame dried flask. Anhydrous DMF (5.0 mL) was added to the dry reagents and the mixture was heated at 105 °C for 18 h. The reaction mixture was allowed to cool to room temperature. DMF was evaporated under reduce pressure to afford residue which was dissolved in ethyl acetate (50 mL). The ethyl acetate was washed with water (20 mL), brine (20 mL), and dried over anhydrous MgSO_4_. The organic solvent was removed under vacuum to give desired product **9** (1.2 g, 76 %) as a brown solid, which was used without further purification. FAB MS calculated C_49_H_51_Br_2_N_5_O_2_Si: m/z = 929.9. Found : m/z 929.9 (M^+^). mp: 104-105 °C. ^1^HNMR (500 MHz, CDCl_3_). δ (ppm): 7.94 (brs, 2H), 7.50-7.49 (m, 2H), 7.40 (d, *J*= 7.5 Hz, 4H), 7.29 (d, *J*= 8.0 Hz, 4H), 7.15 (d, *J*= 8.0 Hz, 2H), 7.01 (d, *J*= 9.0 Hz, 4H), 6.79-6.75 (m, 6H), 3.86 (t, *J*= 6.5 Hz, 4H), 1.74-1.68 (m, 4H), 1.43-1.37 (m, 4H), 1.32-1.27 (m, 8H), 0.87 (t, *J*= 6.7 Hz, 6H); ^13^CNMR (125 MHz, CDCl_3_). δ (ppm):155.9, 150.4, 139.6, 137.6, 136.9, 136.6, 136.5, 132.8, 131.1, 127.4, 126.3, 124.8, 120.7, 120.5, 118.0, 115.3, 115.2, 68.2, 31.5, 29.2, 25.6, 22.5, 14.0; FTIR (Neat) 3329, 2930, 2858, 1590, 1504, 1478, 1239, 1111, 1067, 1009, 809, 732 cm^-1^

**Synthesis of 4-((4-(5-(4-*Tert*-butylphenyl)-1,3,4-oxadiazol-2-yl)phenyl)bis(4-bromophenyl) silyl)-N,N-bis(4-(hexyloxy)phenyl)benzenamine (10).** To a solution of 4-((4-(1H-tetrazol-5- yl)phenyl)bis(4-bromophenyl)silyl)-N,N- bis(4- (hexyloxy)phenyl)aniline **9** (1.2 g, 1.29 mmol) in pyridine (8 mL) was added 4-*tert*butylbenzoyl chloride (0.305 mL, 1.55 mmol). The mixture was stirred and heated at 100 °C for 6 h. The reaction mixture was then cooled to room temperature. Pyridine was evaporated under reduce pressure to afford residue which was dissolved in ethyl acetate (40 mL). The ethyl acetate was washed with water (20 mL), brine (20 mL), and dried over anhydrous MgSO_4_. The organic solvent was removed under vacuum to give residue which was purified by silica gel column chromatography using hexane and ethyl acetate (95:5) as eluent to afford the title compound **10** (0.82 g, 60 %) as a white solid. mp: 150-153 °C. ^1^HNMR (500 MHz, CD_2_Cl_2_). δ (ppm): 8.10 (d, *J*= 8.0 Hz, 2H), 8.04 (d, *J*= 8.5 Hz, 2H), 7.68 (d, *J*= 8.5 Hz, 2H), 7.56 (d, *J*= 8.5 Hz, 2H), 7.53 (d, *J*= 8.0 Hz, 4H), 7.41 (d, *J*= 8.5 Hz, 4H), 7.25 (d, *J*= 8.5 Hz, 2H), 7.07 (d, *J*= 8.5 Hz, 4H), 6.84-6.81 (m, 6H), 3.90 (t, *J*= 6.5 Hz, 4H), 1.76-1.70 (m, 4H), 1.44-1.39 (m, 4H), 1.35 (s, 9H), 1.32-1.30 (m, 8H), 0.88 (t, *J*= 6.5 Hz, 6H); ^13^CNMR (125 MHz, CDCl_3_). δ (ppm):164.8, 164.2, 156.1, 155.4, 150.6, 139.7, 138.8, 137.7, 136.9, 136.7, 132.6, 131.2, 127.5, 126.8, 126.0, 125.0, 121.0, 120.2, 118.0, 115.4, 68.2, 35.1, 31.5, 31.1, 29.2, 25.7, 22.6, 14.0; FTIR (Neat) 2961, 2926, 2857, 1590, 1503, 1478, 1237, 1114, 1067, 1009, 810, 732 cm^-1^.


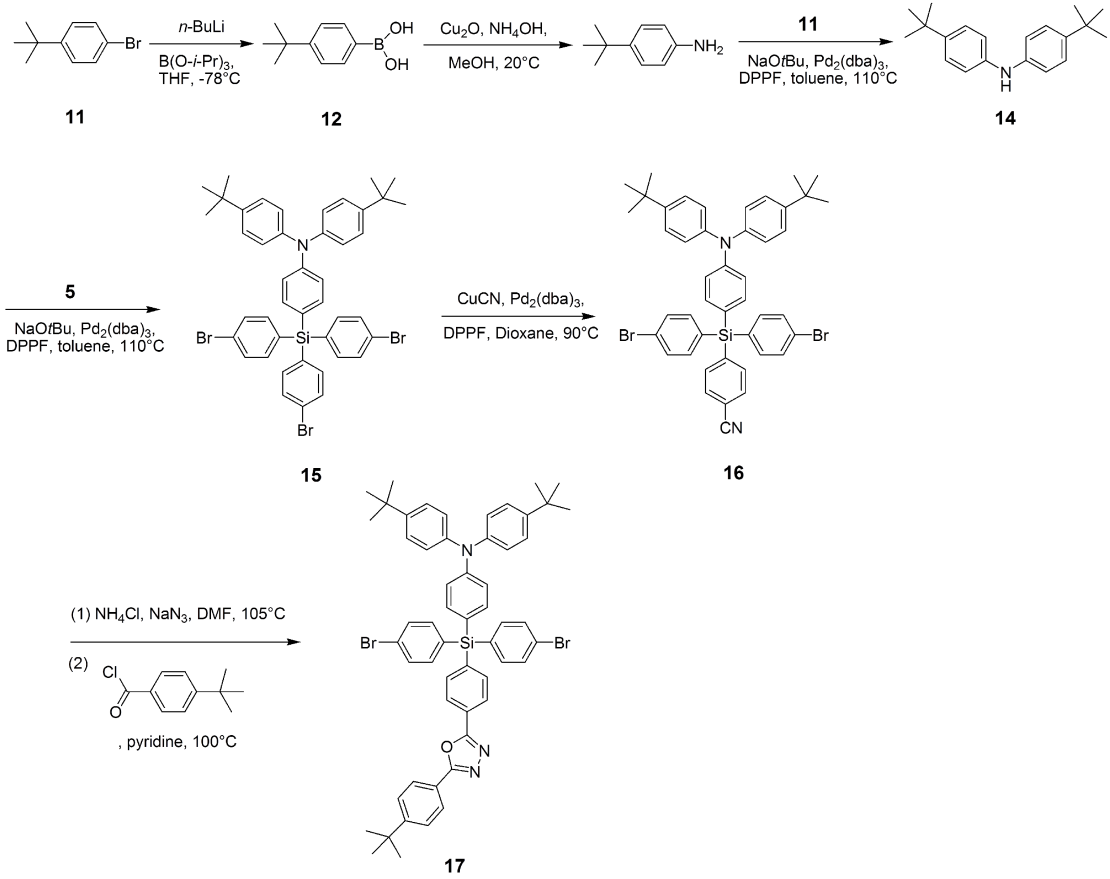


**Supplementary Scheme 3 |** **Synthetic route for monomer 17.**

**Synthesis of 4-*tert*-butylphenylboronic acid (12).** 1-Bromo-4-*tert*-butylbenzene **11** (20.0 g, 93.84 mmol) was dissolved in dry THF (300 mL) and cooled down to -78 °C. *n*-Butyllithium (2.5M in hexane, 41.6 mL, 103.22mmol) was then added dropwisely via a syringe under nitrogen atmosphere, and the mixture was stirred for further 45 min at -78 °C. Triisopropyl borate (26.0 mL, 112.6mmol) was then added dropwisely to the reaction mixture at -78 °C under nitrogen atmosphere. The reaction mixture was allowed to warm up to room temperature over a period of 1 h. After the addition of a saturated aqueous ammonium chloride solution, the mixture was extracted with ethyl acetate (2 x 50 mL). The combined solvents were washed with a saturated aqueous sodium hydrogen carbonate solution (50 mL) and a saturated aqueous sodium chloride solution (50 mL), and dried over anhydrous MgSO_4_. The organic solvent was removed under reduced pressure to give acid **12** (14.5 g, 86.8 %) as a white solid. The product was used without further purification. mp: 194-195 °C (lit.^10^ 191-193 °C); ^1^HNMR (500 MHz, CDCl_3_). δ (ppm): 7.66 (d, *J*= 8.0 Hz, 2H), 7.43 (d, *J*= 8.5 Hz, 2H), 4.54 (s, 2H), 1.31 (s, 9H); ^13^CNMR (125 MHz, DMSO). δ (ppm):152.9, 134.6, 124.6, 34.1, 31.3; FTIR (Neat) 3589, 3449, 2963, 2904, 2868, 1608, 1421, 1284, 1251, 1127, 778, 685 cm^-1^

**Synthesis of 4-*tert*-butylbenzenamine (13)**. 4-*Tert*-butylbenzenamine **13** was prepared using the procedure reported in the literature ^11^ but 4-*tert*-butylphenylboronic acid **12** was used as the starting material. Product **13** was obtained as a brown oil. Yield 50.6 %. GC-MS calculated C_10_H_15_N : m/z = 149.2. Found : m/z 149 (M^+^). ^1^HNMR (500 MHz, CDCl_3_). δ (ppm): 7.17 (d, *J*= 8.0 Hz, 2H), 6.63 (d, *J*= 9.0 Hz, 2H), 3.52 (br s, 2H), 1.26 (s, 9H); ^13^CNMR (125 MHz, CDCl_3_). δ (ppm): 143.6, 141.2, 126.0, 114.8, 33.7, 31.4; FTIR (Neat) 3373, 2962, 2903, 2866, 1610, 1518, 1319, 1269, 1068, 823, 735 cm^-1^

**Synthesis of bis(4-*tert*-butylphenyl)amine (14).** Compound **14** was prepared in a similar process to that of **3** but 4-tert-butylaniline **13** and 1-bromo-4-*tert*-butylbenzene **11** were used as the starting materials. Product **14** was obtained as an orange solid. Yield 62 %. mp: 107-109 °C (lit.^12^ 108-110 °C); ^1^HNMR (500 MHz, CDCl_3_). δ (ppm): 7.26 (d, *J*= 8.0 Hz, 4H), 6.99 (d, *J*= 8.5 Hz, 4H), 5.52 (br s, 1H), 1.29 (s, 18H); ^13^CNMR (125 MHz, CDCl_3_). δ (ppm): 143.5, 140.9, 126.0, 117.4, 34.1, 31.5; FTIR (Neat) 3374, 2962, 2865, 1610, 1520, 1318, 1190, 823, cm^-1^

**Synthesis of N,N-bis(4-*tert*-butylphenyl)-4-(tris(4-bromophenyl)silyl)benzenamine (15).** Compound **15** was prepared in a similar process to that of **3** but bis(4-tert-butylphenyl)- amine **14** and tetrakis(4-bromophenyl)silane **5** were used as the starting materials. White solid. Yield 62 %. FAB MS calculated C_44_H_42_Br_3_NSi : m/z = 853. Found : m/z 853 (M^+^). mp: 135-137 °C. ^1^HNMR (500 MHz, CDCl3). δ (ppm): 7.49 (d, *J*= 8.0 Hz, 6H), 7.35 (d, *J*= 8.5 Hz, 6H), 7.26-7.22 (m, 6H), 7.04 (d, *J*= 8.5 Hz, 4H), 6.72 (d, *J*= 8.0 Hz, 2H), 1.28 (s, 18H); ^13^CNMR (125 MHz, CDCl_3_). δ (ppm):150.0, 146.7, 144.2, 137.7, 136.9, 132.7, 131.2, 126.2, 125.1, 124.9, 122.1, 120.1, 34.3, 31.4; FTIR (Neat) 2962, 2903, 2867, 1587, 1571, 1506, 1322, 1269, 1107, 1067, 1010, 808, 731 cm^-1^

**Synthesis of 4-((4-(Bis(4-*tert*-butylphenyl)amino)phenyl)bis(4-bromophenyl)silyl)benzo- nitrile (16).** Compound **16** was prepared in a similar process to that of **8** but 4-*tert*-butyl-N-(4- *tert*-butylphenyl)-N-(4-(tris(4-bromophenyl)silyl)phenyl)aniline **15** was used as the starting material. White solid Yield 39 %. FAB MS calculated C_45_H_42_Br_2_N_2_Si : m/z = 798.7. Found : m/z 798.7 (M^+^). mp: 110-112 °C. ^1^HNMR (500 MHz, CDCl3). δ (ppm): 7.63-7.58 (m, 4H), 7.51 (d, *J*= 8.5 Hz, 4H), 7.33 (d, *J*= 8.0 Hz, 4H), 7.27 (d, *J*= 8.0 Hz, 4H), 7.21 (d, *J*= 8.0 Hz, 2H), 7.05 (d, *J*= 9.0 Hz, 4H), 6.96 (d, *J*= 9.0 Hz, 2H), 1.28 (s, 18H); ^13^CNMR (125 MHz, CDCl_3_). δ (ppm):150.3, 146.9, 144.1, 141.0, 137.6, 136.9, 136.5, 131.8, 131.4, 131.2, 126.2, 125.3, 125.2, 120.9, 119.9, 118.7, 113.5, 34.4, 31.4; FTIR (Neat) 2962, 2230, 1587, 1571, 1501, 1322, 1105, 1010, 810, 733 cm^-1^

**Synthesis of *N,N*-bis(4-tert-butylphenyl)-4-((4-(5-(4-tert-butylphenyl)-1,3,4-oxadiazol-2- yl)phenyl)bis(4-bromophenyl)silyl)benzenamine (17).** 4-((4-(bis(4-tert-butylphenyl)amino) phenyl)bis(4-bromophenyl)silyl)benzonitrile **16** (1.00 g, 1.25 mmol), sodium azide (0.406 g, 6.25 mmol) and ammonium chloride (0.334 g, 6.25 mmol) were combined in a flame dried flask. Anhydrous DMF (8.0 mL) was added to the dry reagents and the mixture was heated at 105 °C for 18 h. The reaction mixture was allowed to cool to room temperature. DMF was evaporated under reduce pressure to afford residue which was dissolved in ethyl acetate (40 mL). The ethyl acetate was washed with water (15 mL), brine (15 mL), and dried over anhydrous MgSO_4_. The organic solvent was removed under vacuum to give 1.02 g (97 %) which was treated with 4-*tert*butylbenzoyl chloride (0.28 mL, 1.42 mmol) in pyridine at 100 °C for 6 h. The reaction mixture was then cooled to room temperature. Pyridine was evaporated under reduce pressure to afford residue which was dissolved in ethyl acetate (30 mL). The ethyl acetate was washed with water, brine, and dried over anhydrous MgSO_4_. The organic solvent was removed under vacuum to give residue which was purified by silica gel column chromatography using hexane and ethyl acetate (95:5) as eluent to afford the title compound **17** (0.57 g, 50 %) as a white solid. FAB MS calculated C_56_H_55_Br_2_N_3_OSi : m/z = 973.9. Found : m/z 973.8 (M^+^). mp: 160-161 °C. ^1^HNMR (500 MHz, CDCl_3_). δ (ppm): 8.11 (d, *J*= 8.5 Hz, 2H), 8.04 (d, *J*= 8.5 Hz, 2H), 7.67 (d, *J*= 8.5 Hz, 2H), 7.54-7.51 (m, 6H), 7.39 (d, *J*= 8.0 Hz, 4H), 7.29-7.26 (m, 6H), 7.06 (d, *J*= 8.5 Hz, 4H), 6.98 (d, *J*= 9.0 Hz, 2H), 1.35 (s, 9H), 1.28 (s, 18H); ^13^CNMR (125 MHz, CDCl_3_). δ (ppm):164.8, 164.2, 155.5, 150.1, 146.8, 144.2, 138.7, 137.7, 136.9, 136.7, 132.5, 131.3, 126.8, 126.2, 126.1, 125.1, 121.8, 121.0, 120.2, 35.1, 34.3, 31.4, 31.1; FTIR (Neat) 2962, 2903, 2868, 1587, 1571, 1496, 1363, 1269, 1108, 1066, 1010, 733 cm^-1^


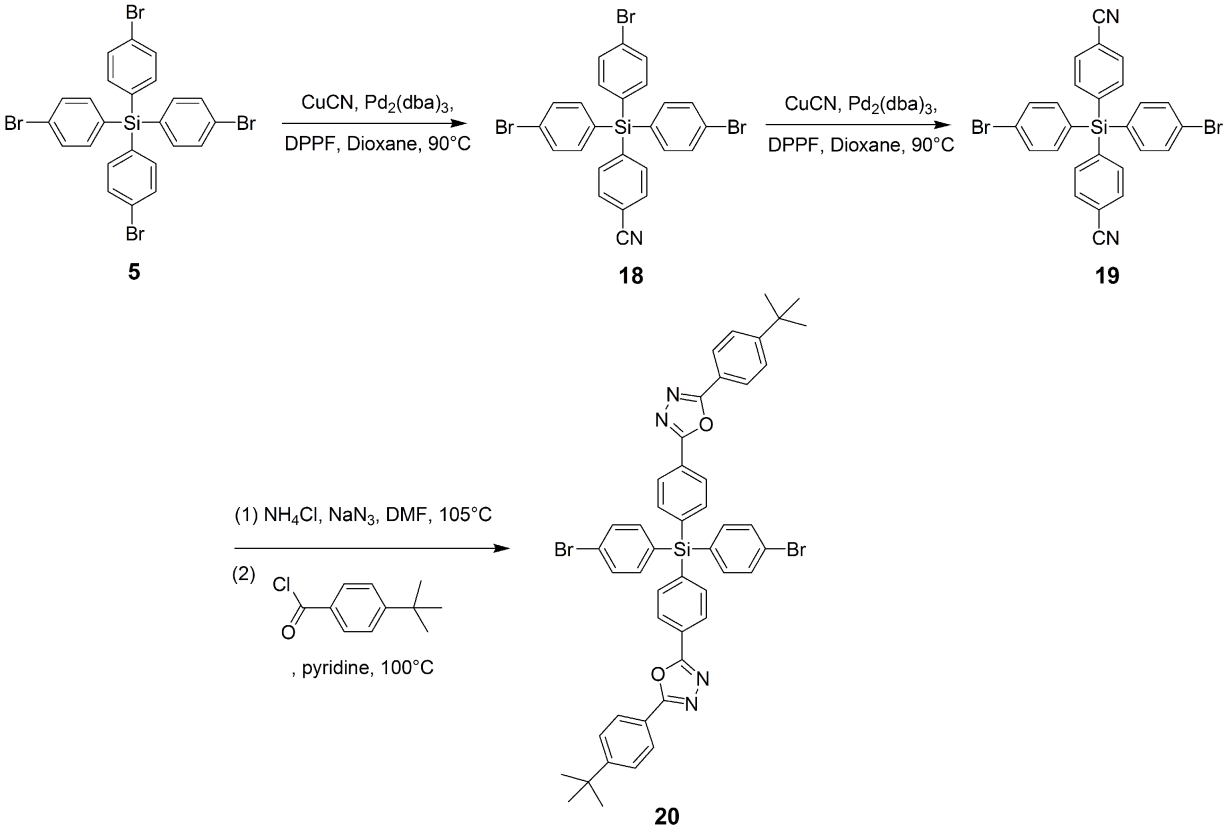


**Supplementary Scheme 4 | Synthetic route for monomer 20.**

**Synthesis of 4-(tris(4-bromophenyl)silyl)benzonitrile (18).** Compound **18** was prepared in a similar process to that of **8** but tetrakis(4-bromophenyl)- silane **5** was used as the starting material. White solid. Yield 47 %. mp: 77-79 °C. ^1^HNMR (500 MHz, CDCl_3_). δ (ppm): 7.64 (d, *J*= 8.0 Hz, 2H), 7.57 (d, *J*= 8.5 Hz, 2H), 7.54 (d, *J*= 8.0 Hz, 6H), 7.30 (d, *J*= 8.5 Hz, 6H); ^13^CNMR (125 MHz, CDCl_3_). δ (ppm):139.6, 137.5, 136.5, 131.7, 131.4, 130.5, 125.8, 118.5, 113.9; FTIR (Neat) 2961, 2230, 1571, 1479, 1378, 1067, 1009, 808, 733 cm^-1^

**Synthesis of 4-(bis(4-bromophenyl)silyl)dibenzonitrile (19)**. Compound **19** was prepared in a similar process to **8** but 4-(tris(4-bromophenyl)- silyl)benzonitrile **18** was used as the starting material. White solid. Yield 37 %. mp: 76-78 °C. ^1^HNMR (500 MHz, CDCl_3_). δ (ppm): 7.67 (d, *J*= 8.0 Hz, 4H), 7.58-7.55 (m, 8H), 7.30 (d, *J*= 8.5 Hz, 4H); ^13^CNMR (125 MHz, CDCl_3_). δ (ppm):138.6, 137.5, 136.5, 131.8, 131.5, 129.6, 126.1, 118.3, 114.3; FTIR (Neat) 2963, 2230, 1571, 1479, 1378, 1067, 1010, 809, 734 cm^-1^

**Synthesis of 2-(4-tert-butylphenyl)-5-(4-((4-(5-(4-tert-butylphenyl)-1,3,4-oxadiazol-2-yl) phenyl)bis(4-bromophenyl)silyl)phenyl)-1,3,4-oxadiazole (20).** Compound **20** was prepared in a similar process to **17** but 4,4'-(bis(4-bromophenyl)- silanediyl)dibenzonitrile **19** was used as the starting material, which was purified by silica gel column chromatography using dichloromethane and ethyl acetate (99:1) as eluent to afford the compound **20** as a white solid. Yield 44 %. FAB MS calculated C_48_H_42_Br_2_N_4_O_2_Si : m/z = 894.1. Found : m/z 894.4 (M^+^). mp: 166-168 °C. ^1^HNMR (500 MHz, CDCl_3_). δ (ppm): 8.16 (d, *J*= 8.0 Hz, 4H), 8.04 (d, *J*= 8.0 Hz, 4H), 7.69 (d, *J*= 8.5 Hz, 4H), 7.57 (d, *J*= 8.5 Hz, 4H), 7.54 (d, *J*= 8.5 Hz, 4H), 7.40 (d, *J*= 8.0 Hz, 4H), 1.35 (s, 18H); ^13^CNMR (125 MHz, CDCl_3_). δ (ppm):164.9, 164.0, 155.5, 137.7, 137.0, 136.7, 131.6, 131.0, 126.8, 126.3, 126.1, 125.7, 125.6, 120.9, 35.1, 31.1; FTIR (Neat) 2963, 2904, 2868, 1615, 1571, 1494, 1376, 1010, 810, 732 cm^-1^


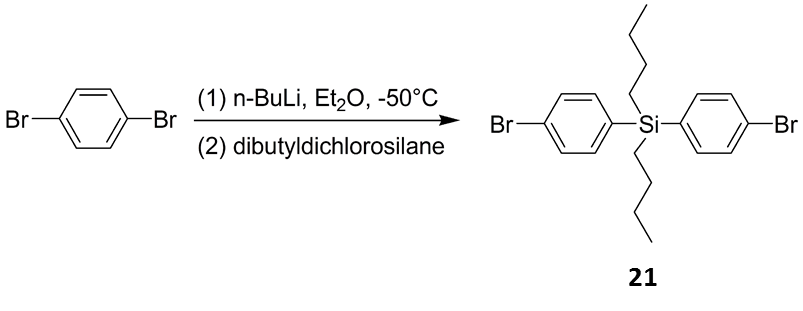


**Supplementary Scheme 5 |** **Synthetic route for monomer 21.**

**Synthesis of bis(4-bromophenyl)dibutylsilane (21).** 1,4-Dibromobenzene (4.72 g, 20 mmol) was dissolved in anhydrous ethyl ether (60 mL), and the solution was cooled to -50°C. 8.4 mL of n-BuLi (2.5 M in hexane, 21 mmol) was then added dropwisely, and the solution was stirred at -20°C for 1h. Then dibutyldichlorosilane (2.13g, 10 mmol) was added dropwisely via a syringe. The mixture was stirred at -20°C for 1h and at room temperature for 3h. Then, aqueous solution of NH_4_Cl (20 mL) was added dropwisely, added more ethyl ether (50ml), and washed with water and brine, dried over MgSO4, and filtered. The organic solvent was removed by evaporation under reduced pressure to afford the oil-like residue. The crude product was re-dissolved in the mixture of hexane/ethanol, and then the white precipitate at -50°C was obtained. The precipitation was collected by filtration and washed with ethanol to afford monomer **25** (1.91g, 42%) as a white solid. mp: 52-53 °C. ^1^HNMR (500 MHz, CDCl_3_). δ (ppm): 7.46 (d, *J*= 8.5 Hz, 4H), 7.29 (d, *J*= 8.0 Hz, 4H), 1.22-1.34 (m, 8H), 0.99-1.03 (m, 4H), 0.84 (t, *J*= 7Hz, 6H); ^13^CNMR (125 MHz, CDCl_3_). δ (ppm):136.3, 134.9, 131.0, 124.1, 26.6, 25.7, 13.6, 12.0; FTIR (Neat) 2949, 2921, 2967, 1573, 1480, 1376, 1067, 1009, 809, 728 cm^-^


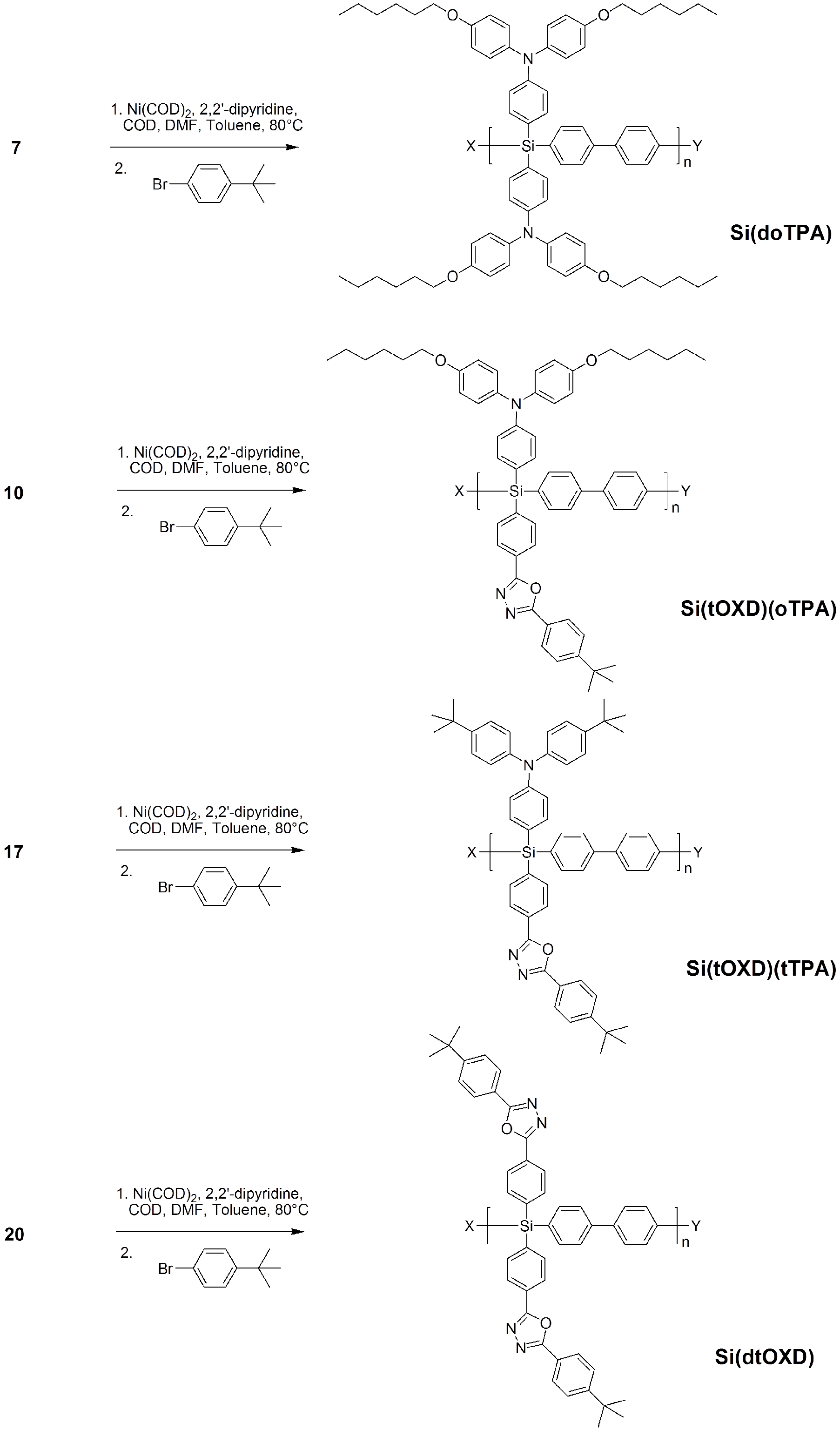


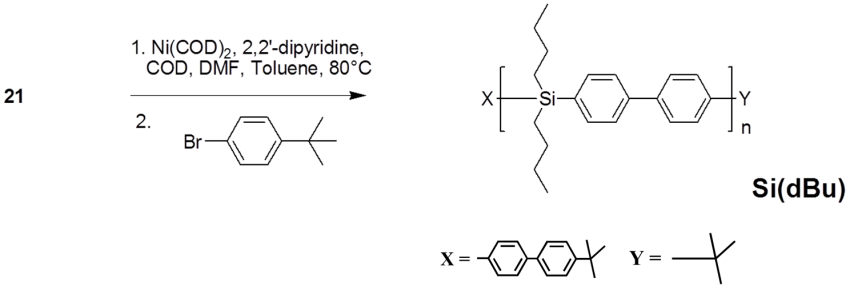


**Supplementary Scheme 6 | Synthetic route for polymers of Si(doTPA), Si(tOXD)(oTPA), Si(tOXD)(tTPA), Si(dtOXD), and Si(dBu).**

**Synthesis of Si(doTPA).** Into a reactor, bis(1,5-cyclooctadiene) nickel (0) (Ni(COD)_2_) (0.185 g, 0.68 mmol), 2,2-bipyridyl (BPY) (0.105 g, 0.68 mmol), 1,5-cyclooctadiene (COD) (0.082 mL, 0.68 mmol) and anhydrous DMF (0.84 mL) were added in a dry box with nitrogen. This mixture was stirred at 80 °C for 30 min to form active catalyst. The monomer **7** (0.5 g, 0.41 mmol) in 2.54 mL of anhydrous toluene was added to the mixture. The polymerization proceeded at 80°C for 4 days in the dry box, and then 1-bromo-4-tert-butylbenzene as end-capping agent (0.007 mL, 0.041 mmol) was added to continually react for 24 h. The resulting polymer was poured into methanol, and stirred for 30 min. The precipitate was collected by filtration and dried and then dissolved in CHCl_3_. Chloroform was washed with water (6 x 50 mL), dried over anhydrous MgSO_4_, and evaporated under reduced pressure. The material was re-dissolved in THF, and again precipitated in methanol.The precipitate was collected by filtration and dried under high vacuum for 24 h. GPC analysis showed its weight-average molecular weight (*Mw*) and polydispersity of 31000 Da and 1.99, respectively, relative to polystyrene standards. ^1^HNMR (500 MHz, CDCl_3_). δ (ppm): 7.65 (d, *J*= 7.5 Hz, 4H), 7.57 (d, *J*= 8.0 Hz, 4H), 7.33 (d, *J*= 8.5 Hz, 4H), 7.04 (d, *J*= 8.5 Hz, 8H), 6.85 (d, *J*= 7.5 Hz, 4H), 6.78 (d, *J*= 9.0 Hz, 8H), 3.88 (t, *J*= 6.2 Hz, 8H), 1.75-1.69 (m, 8H), 1.41-1.39 (m, 8H), 1.30-1.23 (m, 16H), 0.89-0.86 (m, 12H); ^13^CNMR (125 MHz, CDCl_3_). δ (ppm): 155.7, 140.2, 137.1, 136.8, 128.8, 127.2, 126.4, 120.8, 118.5, 115.3, 115.2.

**Synthesis of Si(tOXD)(oTPA).** Prepared according to the method for **Si(doTPA)** by using **10** as monomer. GPC analysis showed its weight-average molecular weight (*Mw*) and polydispersity of 48000 Da and 2.57, respectively, relative to polystyrene standards. ^1^HNMR (500 MHz, CDCl_3_). δ (ppm): 8.10 (d, *J*= 8.0 Hz, 2H), 8.02 (d, *J*= 8.5 Hz, 2H), 7.76 (d, *J*= 8.0 Hz, 2H), 7.67 (d, *J*= 8.0 Hz, 4H), 7.64 (d, *J*= 8.0 Hz, 4H), 7.50 (d, *J*= 8.5 Hz, 4H), 7.35 (d, *J*= 8.5 Hz, 2H), 7.07 (d, *J*= 8.5 Hz, 4H), 6.88 (d, *J*= 8.0 Hz, 2H), 6.78 (d, *J*= 9.0 Hz, 4H), 3.88 (t, *J*= 6.0 Hz, 4H), 1.74-1.69 (m, 4H), 1.41-1.39 (m, 4H), 1.34-1.26 (m, 17H), 0.85 (t, *J*= 6.5 Hz, 6H); ^13^CNMR (125 MHz, CDCl_3_). δ (ppm): ^13^CNMR (125 MHz, CDCl_3_). δ (ppm): 164.7, 164.4, 155.9, 155.4, 150.3, 142.0, 140.0, 139.9, 137.1, 136.9, 136.8, 133.2, 127.4, 126.8, 126.7, 126.0, 125.9, 124.8, 121.7, 121.1, 118.2, 115.2,68.2, 35.1, 31.5, 31.1, 29.2, 25.7, 22.6, 14.0.

**Synthesis of Si(tOXD)(tTPA).** Prepared according to the method for **Si(doTPA)** by using **17** as monomer. GPC analysis showed its weight-average molecular weight (*Mw*) and polydispersity of 77000 Da and 3.40, respectively, relative to polystyrene standards. ^1^HNMR (500 MHz, CDCl_3_). δ (ppm): 8.11 (d, *J*= 8.0 Hz, 2H), 8.02 (d, *J*= 8.5 Hz, 2H), 7.77 (d, *J*= 8.0 Hz, 2H), 7.69 (d, *J*= 7.5 Hz, 4H), 7.64 (d, *J*= 7.5 Hz, 4H), 7.51 (d, *J*= 8.5 Hz, 2H), 7.39 (d, *J*= 8.5 Hz, 2H), 7.26-7.21 (m, 4H), 7.05 (d, *J*= 9.0 Hz, 4H), 7.00 (d, *J*= 8.5 Hz, 2H), 1.32 (s, 9H), 1.26 (s, 18H); ^13^CNMR (125 MHz, CDCl_3_). δ (ppm): ^13^CNMR (125 MHz, CDCl_3_). δ (ppm): 164.7, 164.4, 155.4, 149.8, 146.5, 144.4, 142.0, 139.9, 137.1, 136.9, 136.9, 133.1, 126.8, 126.7, 126.1, 126.0, 125.9, 124.9, 124.8, 123.3, 121.1. 120.4, 35.1, 34.3, 31.4, 31.1.

**Synthesis of Si(dtOXD).** Prepared according to the method for **Si(doTPA)** by using **20** as monomer. GPC analysis showed its weight-average molecular weight (*Mw*) and polydispersity of 12000 Da and 1.40, respectively, relative to polystyrene standards. ^1^HNMR (500 MHz, CDCl_3_). δ (ppm): 8.17-8.14 (m, 4H), 8.05 (m, 4H), 7.80-7.77 (m, 4H), 7.76-7.69 (m, 8H), 7.54-7.50 (m, 4H), 1.35-1.32 (m, 18H); ^13^CNMR (125 MHz, CDCl_3_). δ (ppm): 164.9, 164.2, 155.5, 138.1, 136.9, 136.3, 131.9, 127.0, 126.8, 126.3, 126.2, 126.1, 125.4, 121.0.

**Synthesis of Si(dBu).** Prepared according to the method for **Si(doTPA)** by using **21** as monomer. GPC analysis showed its weight-average molecular weight (*Mw*) and polydispersity of 60000 Da and 1.45, respectively, relative to polystyrene standards. ^1^HNMR (500 MHz, CDCl_3_). δ (ppm): 7.60-7.54 (m, 8H), 1.36-1.35 (m, 8H), 1.11 ((t, *J*= 7Hz, 4H)), 0.86 (t, *J*= 7Hz, 6H). ^13^CNMR (125 MHz, CDCl_3_). δ (ppm): 135.6, 135.3, 126.4, 26.7, 26.0, 13.7,12.4.

**Supplementary References**

1. de Leeuw, D. M., Simenon, M. M. J., Brown, A. R. & Einerhand, R. E. F. Stability of n-type doped conducting polymers and consequences for polymeric microelectronic devices. *Synth. Met*. **87**, 53-59 (1997).
2. (**a**) Dome´nech, A. *et al.* Ship-in-a-bottle synthesis of triphenylamine inside faujasite supercages and generation of the triphenylamminium radical ion. *Tetrahedron* **61**, 791-796 (2005). (**b**) Leung, M.-K. *et al.* The Unusual Electrochemical and Photophysical Behavior of 2,2’-Bis(1,3,4-oxadiazol-2-yl)biphenyls, Effective Electron Transport Hosts for Phosphorescent Organic Light Emitting Diodes. *Org. Lett.* **9**, 235-238 (2007).
3. Tsuchiya, K. *et al.* Synthesis of bipolar charge transporting block copolymers and characterization for organic light-emitting diode. *J. Polym. Sci., Part A: Polym. Chem*. **48**, 1461-1468 (2010).
4. Burkhart, R. D. & Jhon, N. I. Triplet Excimer Formation of Triphenylamine and Related Chromophores in Polystyrene Films. *J. Phys. Chem*. **95**, 7189-7196 (1991).
5. Helander, M. G. *et al.* Chlorinated Indium Tin Oxide Electrodes with High Work Function for Organic Device Compatibility. *Science* **332**, 944-947 (2011).
6. Baldo, M. A., O'Brien, D. F., Thompson, M. E. & Forrest, S. R. Excitonic singlet-triplet ratio in a semiconducting organic thin film. *Phys. Rev. B* **60**, 14422 (1999).
7. Liao, J.-L. et al. Investigating Side Chain Mediated Electroluminescence from Carbazole-Modified Polyfluorene. *J. Phys. Chem. B* **111**, 10379-10385 (2007).
8. Zhang, B. & Wang, Z. Building ultramicropores within organic polymers based on a thermosetting cyanate ester resin. *Chem. Commun*. 5027-5029 (2009).
9. Rao, H., Fu, H., Jiang, Y. & Zhao, Y. Easy Copper-Catalyzed Synthesis of Primary Aromatic Amines by Couplings Aromatic Boronic Acids with Aqueous Ammonia at Room Temperature. *Angew. Chem. Int. Ed*. **48**, 1114-1116 (2009).
